# Synthetic Retinoid Kills Drug‐Resistant Cancer Stem Cells via Inducing RAR*γ*‐Translocation‐Mediated Tension Reduction and Chromatin Decondensation

**DOI:** 10.1002/advs.202203173

**Published:** 2022-08-28

**Authors:** Yao Zhang, Qi Dong, Quanlin An, Chumei Zhang, Erfan Mohagheghian, Bing Niu, Feng Qi, Fuxiang Wei, Sihan Chen, Xinman Chen, Anqi Wang, Xin Cao, Ning Wang, Junwei Chen

**Affiliations:** ^1^ Key Laboratory of Molecular Biophysics of the Ministry of Education Laboratory for Cellular Biomechanics and Regenerative Medicine Department of Biomedical Engineering College of Life Science and Technology Huazhong University of Science and Technology Wuhan Hubei 430074 China; ^2^ Institute of Clinical Science Zhongshan Hospital Fudan University 180 Fenglin Road Shanghai 200032 China; ^3^ Department of Mechanical Science and Engineering The Grainger College of Engineering University of Illinois at Urbana‐Champaign Urbana IL 61801 USA; ^4^ School of Life Sciences Shanghai University 99 Shangda Road Shanghai 200444 China

**Keywords:** cancer stem cells, cell apoptosis, chromatin decondensation, drug efficacy, tension force

## Abstract

A recently developed synthetic retinoid abrogates proliferation and induces apoptosis of drug‐resistant malignant‐cancer‐stem‐cell‐like cells. However, the underlying mechanisms of how the synthetic retinoid induces cancer‐stem‐cell‐like cell tumor‐repopulating cell (TRC) apoptosis are elusive. Here, it is shown that although the retinoid and conventional anticancer drugs cisplatin, all‐trans retinoic acid, and tazarotene all inhibit cytoskeletal tension and decondense chromatin prior to inducing TRC apoptosis, half‐maximal inhibitory concentration of the retinoid is 20‐fold lower than those anticancer drugs. The synthetic retinoid induces retinoic acid receptor gamma (RAR*γ*) translocation from the nucleus to the cytoplasm, leading to reduced RAR*γ* binding to Cdc42 promoter and Cdc42 downregulation, which decreases filamentous‐actin (F‐actin) and inhibits cytoskeletal tension. Elevating F‐actin or upregulating histone 3 lysine 9 trimethylation decreases retinoid‐induced DNA damage and apoptosis of TRCs. The combinatorial treatment with a chromatin decondensation molecule and the retinoid inhibits tumor metastasis in mice more effectively than the synthetic retinoid alone. These findings suggest a strategy of lowering cell tension and decondensing chromatin to enhance DNA damage to abrogate metastasis of cancer‐stem‐cell‐like cells with high efficacy.

## Graphical abstract

Targeted therapy is one of the primary treatments for cancer, but resistance to anticancer drugs hampers their application to many types of malignant tumors. It is shown that synthetic retinoid WYC‐209 overcomes the limitation of conventional anticancer drugs by activating retinoic acid receptor gamma exit from nucleus to induce tension‐abrogation‐mediated chromatin decondensation and DNA damage to effectively kill malignant tumors.

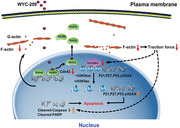

## 1 Introduction

Targeted therapy is one of the frequently used treatments for cancer,^[^ ^1^ ^]^ but resistance to anticancer drugs by cancer cells hampers their effectiveness in treating many types of malignant tumors.^[^ ^2^ ^]^ Cancer stem cells (CSCs) or tumor‐initiating cells (TICs) are a self‐renewing, highly tumorigenic subpopulation of tumor cells^[^ ^3^ ^]^ that are believed to be responsible for tumor initiation, progression, invasion, and metastasis^[^ ^4^ ^]^ and are speculated to be key players in cancer relapse after chemotherapy.^[^ ^4^, ^5^ ^]^ Thus developing novel targeted therapeutic drugs or enhancing inhibitory efficiency of conventional drugs to abrogate CSCs or TICs is essential in cancer research and clinical applications.

We have published a mechanical method for selecting and growing tumorigenic cells from various cancer cell lines and primary cancer cells by culturing single cancer cells in soft fibrin gels.^[^ ^6^ ^]^ The selected cancer cells display high self‐renewal ability and are resistant to chemotherapeutic drugs such as cisplatin and doxorubicin.^[^ ^6^ ^]^ Remarkably, when injected selected cancer cells into tail veins, as few as 10 of such cells can generate distant metastatic colonization in immune‐competent mice^[^ ^6^ ^]^ and even 5 of such cells are able to generate primary tumors in mice.^[^ ^7^ ^]^ We thus functionally define these soft‐fibrin‐gel‐selected cancer cells as tumor‐repopulating cells (TRCs), which are distinct from CSCs or TICs that are selected by stem cell markers. These TRCs express high levels of self‐renewing gene *Sox2* and exhibit low levels of histone 3 lysine residue 9 (H3K9) methylation.^[^ ^8^ ^]^

All‐trans‐retinoic acid (ATRA) and its retinoic analogs such as tazarotene regulate differentiation, proliferation, and apoptosis of various cancer cells, emerging as one of targeted anticancer strategies.^[^ ^9^ ^]^ Cisplatin is a platinum‐based chemotherapy compound, which has been one of the most active clinical drug classes for the treatment of a variety of solid tumors,^[^ ^10^ ^]^ but TRCs exhibit resistance to cisplatin that fails to inhibit growth of TRCs even at high concentrations.^[^ ^6^, ^11^ ^]^ To overcome the drug resistance by TRCs, we have synthesized, screened, and characterized a novel retinoid, named WYC‐209 (molecular weight (MW)  =  368.1 Da), which effectively inhibits proliferation of TRCs and induces apoptosis of multiple cancer cell lines in culture and inhibits lung metastasis of murine melanoma TRCs in immune‐competent mice with no apparent toxicity.^[^ ^11^ ^]^ However, the underlying mechanisms of how WYC‐209 induces apoptosis remain elusive. Furthermore, physical features of tumors are known to regulate tumor progression^[^ ^12^ ^]^ but the relationship between mechanical features of TRCs and drug resistance is not understood.

In this study, we show that conventional drugs cisplatin, ATRA, and tazarotene and the novel retinoid WYC‐209 all induce TRC apoptosis via decreasing cytoskeleton to decondense chromatin. However, the reason why WYC‐209 is more effective than other anticancer drugs is that it can induce retinoic acid receptor gamma (RAR*γ*) translocation from the nucleus to the cytoplasm at a much lower concentration, leading to Cdc42 downregulation and thus reduction of filamentous‐actin (F‐actin) and tractions. This is accompanied by lowering of H3K9 methylation and elevation of H3K9 acetylation to decondense chromatin of TRCs to increase DNA damage by the synthetic retinoid. The combinatorial treatment of WYC‐209 with chaetocin, an inducer of H3K9 demethylation and chromatin decondensation enhances inhibition of lung metastasis of melanoma TRCs in immune‐competent mice.

## 2 Results

### 2.1 Retinoid WYC‐209 Is Highly Potent in Inducing TRC Apoptosis

**Figure 1:**
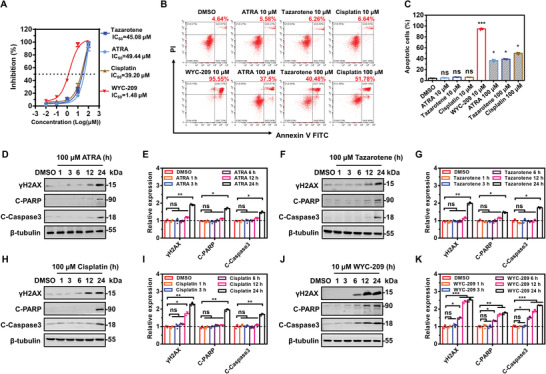
Retinoid WYC‐209 is highly potent in inducing tumor cell apoptosis. A) The IC_50_ values of conventional drugs (all‐trans retinoic acid (ATRA), tazarotene, or cisplatin) or WYC‐209 for B16‐F1 TRCs were determined in the MTT assay after treatment for 48 h. B,C) B16 TRCs were treated with ATRA, tazarotene, cisplatin at 10 and 100 µm and WYC‐209 at 10 µm for 24 h, then the cell apoptotic ratio analyzed by FITC–Annexin‐V and PI apoptosis detection kit. Representative images of flow cytometry data (B) and quantification data of apoptotic cells ratio (C). Apoptotic cells (%) = 100% − (Annexin‐V^−^ PI^−^)%. D–K) Representative western blot images (D, F, H, J) and quantification (E, G, I, K) of relative *γ*H2AX, C‐PARP, and C‐Caspase3 expression of B16 TRCs treated with 100 µm ATRA, 100 µm tazarotene, 100 µm cisplatin, or 10 µm WYC‐209 for various durations. Mean ± s.e.m.; three independent experiments. One‐way ANOVA testing followed by a Tukey post‐hoc test when appropriate was used for statistics. *p < 0.05; **p < 0.01; ***p < 0.001; ns = not significantly different.

3‐(4, 5‐Dimethylthiazol‐2‐yl)‐2, 5‐diphenyltetrazolium bromide (MTT) assays were utilized to quantify half‐maximal inhibitory concentration (IC_50_) of various molecules on TRCs. IC_50_ of WYC‐209 was more than 20‐fold lower than conventional anticancer drugs cisplatin, tazarotene, and ATRA (**Figure** **1**A); 10 µm of conventional anticancer drugs were not able to induce any TRC apoptosis. At 100 µm, 30–50% of TRCs became apoptotic when treated with ATRA, tazarotene, or cisplatin; in sharp contrast, only 10 µm of WYC‐209 was able to induce apoptosis of >95% of TRCs (Figure 1B,C). To determine how these cells became apoptotic, DNA damage and apoptosis‐associated markers phospho‐histone 2AX (*γ*H2AX), cleaved‐Caspase3 (C‐Caspase3), and cleaved poly(adenosine diphosphate (ADP)‐ribose) polymerase (C‐PARP) were assayed. These 3 markers started to express only after 12 h treatment by the conventional anticancer drugs (ATRA, tazarotene, and cisplatin) at 100 µm, whereas they appeared 6 h after 10 µm WYC‐209 treatment (Figure 1D–K), suggesting that WYC‐209 is much more potent than the conventional anticancer drugs in inducing DNA damage and cell apoptosis.

### 2.2 Retinoid WYC‐209 Inhibits F‐Actin and Tension Force before Inducing Apoptosis

**Figure 2:**
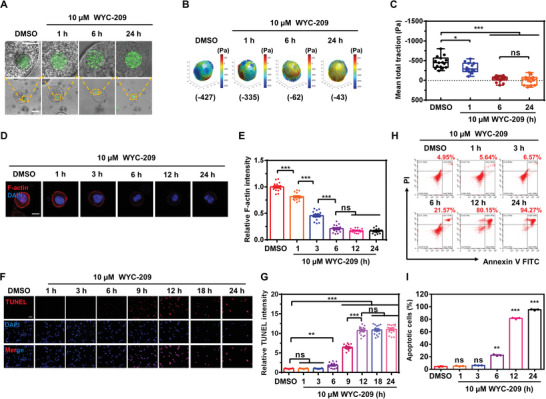
Retinoid WYC‐209 inhibits F‐actin and tension before apoptosis occurs. Representative images of the elastic round microgels embedded in 3D colonies A) of B16‐F1 melanoma tumor‐repopulating cells (TRCs) and B) of 3D traction maps treated with 10 µm WYC‐209 at 0 (DMSO control), 1, 6, and 24 h. The top images in (A) are enlarged images from the respective bottom images. Scale bars in (A), top, 25 µm; bottom, 50 µm. The blue colors in (B) represent compressive stresses and red colors represent tensile stresses. C) Quantification data of mean tractions. Mean ± s.e.m.; *n* = 15 microgels in each condition; three independent experiments. Negative values of tractions indicate that the monodisperse elastic round microgels (≈25 mm) are compressed by actomyosin‐dependent tensile stresses of the surrounding tumor cells.^[^ ^13^ ^]^ D) Representative images of F‐actin distribution in a single TRC colony treated with WYC‐209 for different durations. B16 TRCs: B16‐F1 cells were cultured in 90 Pa fibrin gels for 3 days and then recultured on 2D 90 Pa fibrin gels. F‐actin was stained by phalloidin (red). Nucleus was stained by DAPI (blue). DMSO: medium with 0.1% DMSO. Scale bar: 10 µm. E) Relative F‐actin intensity. Mean ± s.e.m.; *n* = 15 cells for each condition; three separate experiments. F,G) Representative images (F) of immunofluorescence of terminal deoxynucleotidyl transferase dUTP nick end labeling (TUNEL, an assay for measuring cell apoptosis) and quantification (G) of relative TUNEL intensity of B16 TRCs treated with 10 µm WYC‐209. Scale bar: 50 µm. Mean ± s.e.m.; *n* = 15 cells for each condition; three separate experiments. H,I) B16 TRCs were treated with 10 µm WYC‐209 for different time points and the cells apoptotic ratio analyzed by FITC–Annexin‐V and PI apoptosis detection kit. Representative images of flow cytometry data (H) and quantification data of apoptotic cells ratio (I). Apoptotic cells (%) = 100% − (Annexin‐V^−^ PI^−^)%. Mean ± s.e.m.; three separate experiments. One‐way ANOVA testing plus Tukey post‐hoc correction when appropriate was used for statistics. *p < 0.05; **p < 0.01; ***p < 0.001; ns = not significantly different.

Cellular traction is a fundamental mechanical function of live cells. Arg–Gly–Asp‐coated elastic round microgels (ERMGs) were used to quantify tractions in a 3D tumor cell colony.^[^ ^13^ ^]^ As the tumor cells attached to the ERMG and generated tractions onto the microgel, they exerted the compressive stresses onto the microgel (**Figure** **2**A,B). Compressive tractions of TRCs substantially decreased 1 h and completely disappeared 6 h after 10 µm WYC‐209 treatment (Figure 2B,C). Furthermore, F‐actin in TRCs decreased by ≈20% 1 h and by ≈80% 6 h after WYC‐209 treatment (Figure 2D,E). By contrast, TRCs became apoptotic at later times: no apoptosis was observed 3 h (Figure 2F,G), 20% of cells became apoptotic 6 h, and over 80% of cells died 12 h after 10 µm WYC‐209 treatment (Figure 2H,I), consistent with time course of expression of apoptosis‐associated proteins (Figure 1J,K). The dose‐dependent effect of WYC‐209 on tractions was observed: 1.0 µm WYC‐209 did not decrease TRC tractions, but 5.0 and 10 µm WYC‐209 completely abrogated TRC tractions (Figure S1A–C, Supporting Information). F‐actin staining data were consistent with the traction results (Figure S1D,E, Supporting Information). However, 5.0 µm WYC‐209 for 24 h only induced apoptosis in ≈20% of TRCs (Figure S1F,G, Supporting Information) and slight elevation of C‐Caspase 3 expression, much lower than 10 µm WYC‐209 did (Figure S1H,I, Supporting Information). In sharp contrast, the conventional anticancer drugs ATRA, tazarotene, and cisplatin at 10 µm for 24 h did not induce F‐actin depolymerization (Figure S2A,B, Supporting Information) nor did they induce apoptosis (Figure 1B,C). Only after 12 h treatment of 100 µm of these conventional drugs, F‐actin began to decrease (by ≈30%) (Figure S2C,D, Supporting Information). Taken together, WYC‐209 downregulates TRC tractions before inducing apoptosis and is more efficacious than the conventional anticancer drugs.

### 2.3 Modulating F‐Actin and Cdc42 Regulates Retinoid‐Induced TRC Apoptosis

**Figure 3:**
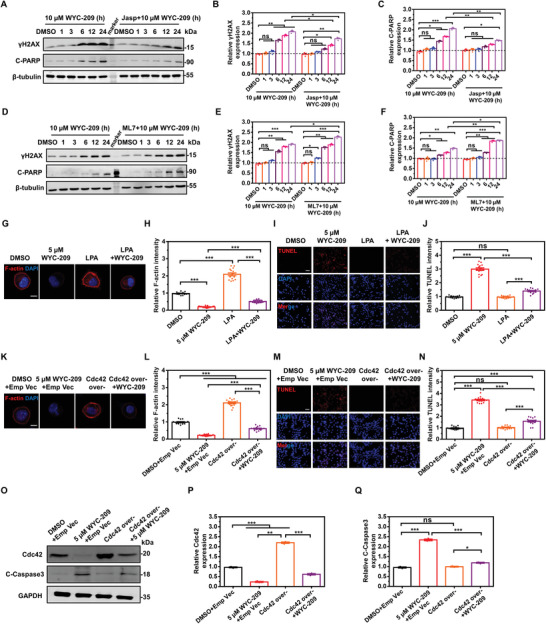
Modulating F‐actin and Cdc42 regulates retinoid‐induced TRC apoptosis. A) Representative western blot images and quantification B,C) of relative *γ*H2AX and C‐PARP expression of B16‐F1 TRCs treated with 10 µm WYC‐209 pretreated without or with Jasplakinolide (Jasp, which induces actin polymerization and stabilization, 3.0 µm for 3 h). Mean ± s.e.m.; three independent experiments. D) Representative western blot images and quantification E,F) of relative *γ*H2AX and C‐PARP expression of B16‐F1 TRCs treated with 10 µm WYC‐209 pretreated with or without ML7 (15 µm for 4 h). Mean ± s.e.m.; three independent experiments. G–J) B16‐F1 was cultured in 90 Pa fibrin gels for 3 days and then recultured on 2D 90 Pa fibrin gels followed by various treatments: DMSO: 0.1% DMSO; 5.0 µm WYC‐209: 5.0 µm WYC‐209 for 24 h; LPA: 10 µm lysophosphatidic acid (LPA) (a drug of activating Rho kinase and promoting actin stress fiber formation) for 4 h; LPA + WYC‐209: B16 TRCs were pretreated with 10 µm LPA for 4 h, washed them out, then treated with 5.0 µm WYC‐209 for another 20 h. Nucleus was stained by DAPI (blue). Representative images (G) of F‐actin and quantification of relative F‐actin (H) intensity. Scale bar: 10 µm. Mean ± s.e.m.; *n* = 15 samples; three independent experiments. Representative images (I) of TUNEL staining and quantification of relative TUNEL intensity (J). Scale bar: 50 µm. Mean ± s.e.m.; *n* = 15 cells for each condition; three separate experiments. K–N) B16‐F1 TRCs were transfected with pcDNA3.1–GFP vector (Emp Vec) or pcDNA3.1–Cdc42 plasmid (Cdc42 over‐) in DMSO (medium with 0.1% DMSO) or 5.0 µm WYC‐209 for 24 h. Representative images of F‐actin (K) and quantification of relative F‐actin (L) intensity. Scale bar: 10 µm. Mean ± s.e.m.; *n* = 15 samples; three independent experiments. Representative images of TUNEL (M) and quantification of relative TUNEL intensity (N). Scale bar: 50 µm. Mean ± s.e.m.; *n* = 15 cells for each condition; three separate experiments. O–Q) Representative western blot images (O) and quantification of relative proteins expression (P, Q) for B16 TRCs transfected with pcDNA3.1–Cdc42 plasmid or pcDNA3.1–GFP (Emp Vec) under DMSO (dimethyl sulfoxide, 0.1%) or 5.0 µm WYC‐209 for 24 h. Mean ± s.e.m.; three separate experiments; one‐way ANOVA testing plus Tukey post‐hoc correction when appropriate was used for statistics. *p < 0.05; **p < 0.01; ***p < 0.001; ns = not significantly different.

It is known that TRCs are undifferentiated cancer‐stem‐cell‐like cells.^[^ ^6^, ^8^ ^]^ TRCs were softer and generated lower tractions than differentiated melanoma cells cultured on rigid dish (Figure S3A,B, Supporting Information), consistent with the published results.^[^ ^6^, ^8^ ^]^ Compared with control B16‐F1 cells cultured on the rigid dish, TRCs exhibited a lower level of F‐actin intensity (Figure S3C,D, Supporting Information) and a lower expression of phosphorylated myosin light‐chain 2 (p‐MLC2) (Figure S3E,F, Supporting Information). WYC‐209 treatment for 6 h did not induce apoptosis of the differentiated melanoma cells (B16 Ctrs) and only ≈50% of these cells became apoptotic after 12 h treatment (Figure S3I,J, Supporting Information), thus less sensitive to the retinoid in comparison to TRCs (Figure 2H,I). Since F‐actin and tractions might be important for the differential biological responses between the differentiated melanoma cells and TRCs, we modulated F‐actin in TRCs. Increasing F‐actin with Jasplakinolide (Jasp) and treating with WYC‐209 produced less *γ*H2AX and C‐PARP in TRCs than with WYC‐209 alone (**Figure** **3**A–C). Decreasing traction with myosin light chain kinase inhibitor ML7 and then treating with WYC‐209 induced more *γ*H2AX and C‐PARP in TRCs than with WYC‐209 alone (Figure 3D–F). Similar results were observed with the conventional anticancer drugs using a combination of F‐actin or myosin‐II modulators and the retinoid (Figure S4A–D, Supporting Information). Because 5.0 µm WYC‐209 resulted in differential effects between TRCs and differentiated melanoma cells, we used this dose in the following experiments. Treating with 5.0 µm WYC‐209 alone decreased F‐actin in TRCs by 80% and treating with RhoA activator lysophosphatidic acid (LPA) increased F‐actin (Figure 3G,H). Pretreating with LPA and then with WYC‐209 dampened the effect of WYC‐209 in lowering the F‐actin (Figure 3G,H). Importantly, pretreating with LPA then with WYC‐209 abrogated the inhibition effect of WYC‐209 in TRCs (Figure 3I,J). We then used a strategy to inhibit F‐actin in the differentiated stiff B16 cells that are known to generate higher tractions.^[^ ^6^ ^]^ Inhibiting F‐actin with latrunculin A (Lat A) and then with WYC‐209 decreased F‐actin dramatically (Figure S5A,B, Supporting Information) and increased apoptosis in these cells, more than with WYC‐209 alone (Figure S5C,D, Supporting Information). Together, these results suggest that modulating F‐actin and/or altering myosin‐II‐mediated traction regulate apoptotic efficiency by the retinoid.

It has been shown that Cdc42 mediates cell stiffening and F‐actin accumulation of TRCs.^[^ ^8^ ^]^ TRCs expressed lower levels of Cdc42 protein than the differentiated melanoma cells (Figure S3G,H, Supporting Information). Depleting Cdc42 together with WYC‐209 treatment decreased F‐actin and increased apoptosis in the differentiated melanoma cells when compared with the synthetic retinoid alone (Figure S5E–K, Supporting Information). By contrast, overexpressing Cdc42 together with WYC‐209 treatment rescued the cells from the WYC‐209 inhibitory effect on F‐actin and dramatically decreased apoptosis (both terminal deoxynucleotidyl transferase 2'‐deoxyuridine 5'‐triphosphate (dUTP) nick end labeling (TUNEL) and C‐Caspase 3 assays) of these cells (Figure 3K–Q). Together these results suggest that WYC‐209 induces apoptosis via the Cdc42‐dependent F‐actin pathway.

### 2.4 WYC‐209 Induces RAR*γ* Translocation to Decrease Its Binding to Cdc42 Promoters

**Figure 4:**
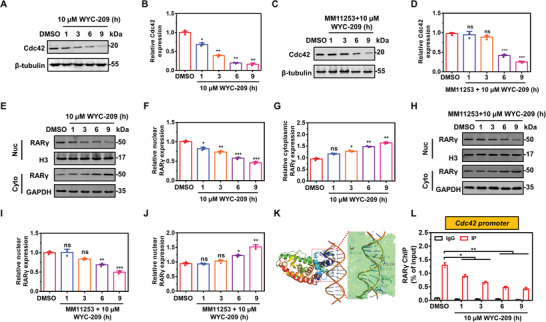
WYC‐209 induces RAR*γ* translocation to decrease its binding to Cdc42 promoters. A,B) Representative western blot images (A) and quantification (B) of relative Cdc42 expression of B16‐F1 TRCs treated with 10 µm WYC‐209 for various durations. Mean ± s.e.m.; three independent experiments. C,D) B16‐F1 TRCs pretreated with MM11253 (RAR*γ* antagonist) then treated with 10 µm WYC‐209 for various time points. Representative western blot images (C) of Cdc42 expression and quantification level (D) of relative Cdc42 expression. Mean ± s.e.m.; three independent experiments. E–G) Representative western blot images (E) of RAR*γ* and quantification data of relative RAR*γ* level in nucleus (F) and cytoplasm (G) of B16‐F1 TRCs treated with 10 µm WYC‐209 for various time points. GAPDH and H3 are used as controls for cytoplasm and nucleus, respectively. Mean ± s.e.m.; three separate experiments. H–J) B16‐F1 TRCs pretreated with MM11253 (RAR*γ* antagonist) then treated with 10 µm WYC‐209 for various time points. Representative western blot images (H) of RAR*γ* expression and quantification level of relative RAR*γ* expression in nucleus (I) and cytoplasm (J). Mean ± s.e.m.; three independent experiments. K) The crystal structure of RAR*γ* was obtained from the protein data bank (PDB) (6FX0) database. The simulated interaction between Cdc42 promoter and RAR*γ* was performed by Discovery Studio 2018, exhibiting tight binding between the two. L) ChIP analysis of RAR*γ* level binding to Cdc42 promoters of B16‐TRCs treated with 10 µm WYC‐209 for various time points. Mean ± s.e.m.; three independent experiments. One‐way ANOVA testing plus Tukey post‐hoc correction when appropriate was used for statistics. *p < 0.05; **p < 0.01; ***p < 0.001; ns = not significantly different.

Next, we examined how WYC‐209 decreases Cdc42 levels. It has been reported that WYC‐209 binds to RAR*γ* with high affinity.^[^ ^11^ ^]^ The messenger RNA (mRNA) expression levels of RAR*γ* were higher than those of RAR*α* and RAR*β* in B16‐F1 TRC (Figure S6A, Supporting Information). Silencing RAR*γ* led to the most dramatic rescuing effect on WYC‐209‐induced apoptosis of the three isoforms (Figure S6B–D, Supporting Information). These results suggest that WYC‐209 selectively targeted on TRC primarily via RAR*γ*, consistent with the previous reports that RAR*γ* acts as an oncogene in regulating cell fate in melanoma cells.^[^ ^14^, ^15^ ^]^ Pretreating TRCs with the specific RAR*γ* antagonist MM11253 delayed WYC‐209‐mediated Cdc42 downregulation (**Figure** **4**A–D). Although WYC‐209 did not change expressions of RARs (*α*, *β*, or *γ*) in TRCs within 24 h (Figure S7, Supporting Information), it induced translocation of RAR*γ* from the nucleus to the cytoplasm (Figure 4E–G). MM11253 delayed RAR*γ* translocation (Figure 4H–J). Molecular dynamics simulation of 3D binding of RAR*γ* at the promoter site of Cdc42 revealed the specific tight binding between the two (Figure 4K). Chromatin immunoprecipitation (ChIP) data revealed that WYC‐209 indeed decreased the amount of RAR*γ* recruited to Cdc42 promoter sites in a time‐dependent manner (Figure 4L). Reporter assays show that RAR*γ* increases Cdc42 expression via binding to Cdc42 promoter, which is inhibited by WYC‐209 (Figure S8, Supporting Information), consistent with ChIP data. Meanwhile, the RAR*γ* translocation rate of control cells induced by 10 µm WYC‐209 was slower than that of TRCs (Figure S9, Supporting Information). By contrast, 10 µm conventional drugs (ATRA, tazarotene, and cisplatin) did not induce RAR*γ* translocation from the nucleus to the cytoplasm (Figure S10A,B, Supporting Information), consistent with the finding that 10 µm conventional drugs did not inhibit F‐actin (Figure S2A,B, Supporting Information). Together, these results suggest that WYC‐209 induces apoptosis more efficiently than the conventional anticancer drugs because it induces RAR*γ* translocation from the nucleus to the cytoplasm more efficiently, which in turn decreases RAR*γ* recruitment to Cdc42 promoters to downregulate Cdc42 expression.

### 2.5 Cdc42 Promotes Chromatin Condensation via Regulating Histone 3 Lysine 9 Trimethylation (H3K9me3) and H3K9 Acetylation (H3K9ac)

**Figure 5:**
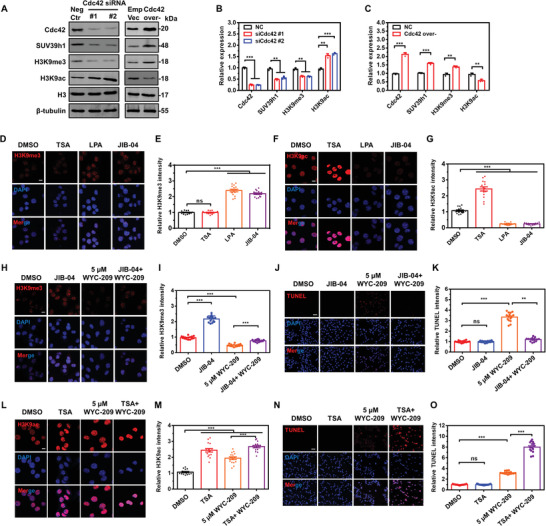
Cdc42 promotes chromatin condensations in tumor cells. A) Representative western blot images of Cdc42, SUV39h1, H3K9me3, and H3K9ac expressions in TRCs after silencing or overexpressing Cdc42. Neg Ctr: B16 TRCs transfected with scrambled siRNA; #1 and #2: B16 TRCs transfected with siRNA #1 or #2 of Cdc42; Emp Vec: B16 TRCs transfected with pcDNA3.1–GFP vector; Cdc42 over‐: B16 TRCs transfected with pcDNA3.1–Cdc42 plasmid. B,C) Quantification data of relative proteins expressions in (A). Mean ± s.e.m.; three separate experiments. D–G) Representative images of H3K9me3 (D) or H3K9ac (F) staining of B16‐F1 TRCs and quantification of relative H3K9me3 (E) or H3K9ac (G) intensity after various treatments. DMSO: medium with 0.1% DMSO; TSA: trichostatin A, a histone deacetylase inhibitor, 0.5 µm for 4 h; LPA: lysophosphatidic acid, 10 µm for 4 h; JIB‐04: a H3K9 demethylase inhibitor, 3.0 µm for 4 h. Scale bar: 10 µm. Mean ± s.e.m.; *n* = 15 cell samples; three separate experiments. H–K) Relative H3K9me3 and TUNEL levels of B16 TRCs with different treatments. DMSO: medium with 0.1% DMSO; JIB‐04: 3.0 µm for 4 h; 5.0 µm WYC‐209: 5.0 µm WYC‐209 for 24 h; JIB‐04 + WYC‐209: JIB‐04, 3.0 µm for 4 h, then washed them out, and treated with 5.0 µm WYC‐209 for 20 h. Representative images of immunofluorescence of H3K9me3 (H) and TUNEL (J). Nucleus stained with DAPI. Histogram shows quantification of relative H3K9me3 (I) and TUNEL (K) intensity. L–O) Relative H3K9ac and TUNEL levels of TRCs with different treatments. DMSO: medium with 0.1% DMSO; TSA: 0.5 µm for 4 h; 5.0 µm WYC‐209: 5.0 µm WYC‐209 for 24 h; TSA + WYC‐209: TSA, 0.5 µm for 4 h, then washed off them, and treated with 5.0 µm WYC‐209 for 20 h. Representative images of immunofluorescence of H3K9ac (L) and TUNEL (N). Nucleus stained with DAPI. Histogram shows quantification of relative H3K9ac (M) and TUNEL (O) intensity. Mean ± s.e.m.; *n* = 15 samples; three separate experiments. Scale bar in (D, F, H, L): 10 µm. Scale bar in (J, N): 50 µm. Two‐tailed Student's *t*‐test plus Bonferroni correction (only two data groups compared) and one‐way ANOVA testing plus Tukey post‐hoc correction when appropriate were used for statistics. **p < 0.01; ***p < 0.001; ns = not significantly different.

Next, we asked whether Cdc42 regulates WYC‐209‐induced apoptosis by regulating levels of chromatin condensation. Since depleting Cdc42 substantially decreased H3K9me3 and H3K9 methyltransferase (SUV39h1) expression but increased H3K9ac, it decondensed chromatin^[^ ^16^, ^17^ ^]^ (**Figure** **5**A,B). Conversely, overexpressing Cdc42 substantially increased H3K9me3 and SUV39h1 but decreased H3K9ac (Figure 5A,C). However, when SUV39h1 was knocked down, H3K9me3 was decreased and H3K9ac was increased; there was no effect in Cdc42 expression (Figure S11A,B, Supporting Information). These data suggest that lowering F‐actin or cellular tension (i.e., traction) induces chromatin decondensation. Electron microscopy shows that WYC‐209 indeed induced chromatin decondensation (Figure S12, Supporting Information). Together with the results that SUV39h1 decreased only after 3 h of WYC‐209 treatment (Figure S11C,D, Supporting Information), slower than Cdc42 decreased by WYC‐209, these data suggest that WYC‐209 induced chromatin decondensation and Cdc42 might be upstream of H3K9me3 and H3K9ac. Indeed overexpressing Cdc42 increased H3K9me3 expression and slowed down the reduction of H3K9me3 expression by WYC‐209, whereas knocking down Cdc42 decreased H3K9me3 and accelerated H3K9me3 reduction by WYC‐209 (Figure S13A,B, Supporting Information). By contrast, overexpressing Cdc42 or knocking down Cdc42 led to the opposite effects on H3K9ac (Figure S13C,D, Supporting Information). Immunofluorescence stain and western blots show that TRCs exhibited lower levels of H3K9me3 and higher levels of H3K9ac in comparison with control B16 cells (Figure S14, Supporting Information). Treating with LPA to increase F‐actin and treating with JIB‐04 (a H3K9 demethylase inhibitor)^[^ ^18^ ^]^ substantially increased H3K9me3 and decreased H3K9ac, but treating with deacetylase inhibitor trichostatin A (TSA) increased H3K9ac and had no effects on H3K9me3 (Figure 5D–G and Figure S15A,B (Supporting Information)). Furthermore, treating with Lat A to decrease F‐actin or treating with chaetocin (an inhibitor of H3K9me3 methyltransferase SUV39h1)^[^ ^19^ ^]^ dramatically decreased H3K9me3 and increased H3K9ac in control B16 cells, but treating TSA only increased H3K9ac but had no effects on H3K9me3 (Figure S16A–D, Supporting Information), suggesting that chaetocin decondenses chromatins of TRCs via lowering H3K9me3 to elevate H3K9ac. Western blots and electron microscopy images show that treating chaetocin alone could only alter levels of H3K9me3 and H3K9ac to decondense chromatin without inducing apoptosis‐related proteins expression (Figures S17 and S18, Supporting Information), and MTT assays show that chromatin structure regulators or small interfering RNA (siRNA) treatments do not effect cell viability (Figure S19, Supporting Information). We then asked whether regulating chromatin condensation could affect apoptosis‐inducing efficiency of WYC‐209. Treating with JIB‐04 to condense chromatin and then with WYC‐209 decreased the effect of WYC‐209 in lowering H3K9me3 and decreased apoptosis in TRCs (Figure 5H–K and Figure S15C,D (Supporting Information)). Treating with TSA to decondense chromatin and then with WYC‐209 increased the effect of WYC‐209 in elevating H3K9ac and then increased the apoptosis in TRCs (Figure 5L–O and Figure S15E,F (Supporting Information)). In control B16 cells, decreasing H3K9me3 by chaetocin or increasing H3K9ac by TSA promoted WYC‐209 apoptotic effect (Figure S16E–L, Supporting Information). 10 µm conventional anticancer drugs did not inhibit H3K9me3 (Figure S20A,B, Supporting Information), but 100 µm ATRA and tazarotene inhibited H3K9me3 after 12 h and 100 µm cisplatin slightly inhibited H3K9me3 after 6 h (Figure S20C,D, Supporting Information), after decreasing F‐actin (Figure S2C,D, Supporting Information). Moreover, pretreating with chaetocin to downregulate H3K9me3 and then treating with conventional drugs increased expression of cell‐apoptosis‐associated proteins, demonstrated that decondensing chromatin increased the inducing apoptotic efficiency of conventional drugs, which is consistent with WYC‐209 treatment groups combined with chaetocin (Figure S20E–J, Supporting Information). Taken together, these data suggest that anticancer drugs induce apoptosis via regulating chromatin condensation by regulating H3K9me3 and H3K9ac via Cdc42 and WYC‐209 inhibits H3K9me3 better than conventional drugs.

### 2.6 WYC‐209 Induces DNA Damages via H3K9 Acetylation to Decondense Chromatin

**Figure 6:**
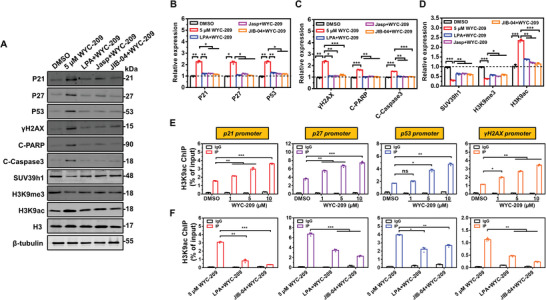
WYC‐209 induces DNA damages via H3K9 acetylation to decondense chromatin. A) Representative western blot images of P21, P27, P53, *γ*H2AX, C‐PARP, C‐Caspase3, H3K9ac, H3K9me3, SUV39h1 expressions of B16 TRCs under various treatments. Various treatments: TRC DMSO: medium with 0.1% DMSO for 24 h; 5.0 µm WYC‐209: 5.0 µm of WYC‐209 for 24 h; LPA + WYC‐209: LPA, pretreated with 10 µm for 4 h, then washed out and treated with 5.0 µm WYC‐209 for 20 h; Jasp + WYC‐209: pretreated with Jasp (which induces actin polymerization and stabilization, 3.0 µm for 4 h), then washed out and treated with 5.0 µm WYC‐209 for 20 h; JIB‐04 + WYC‐209: JIB‐04, pretreated with 3.0 µm for 4 h, then washed out and treated with 5.0 µm WYC‐209 for 20 h. B–D) Quantification data of relative proteins expression in (A). Mean ± s.e.m.; three independent experiments. E) ChIP assays were performed using normal rabbit IgG (negative control, 2 µg per IP) and H3K9ac antibody (2 µg per IP) on sheared chromatin from 3 million B16 TRCs treated with 0.1% DMSO, WYC‐209 of 1.0, 5.0, 10 µm for 24 h. F) ChIP assays were performed using normal rabbit IgG (negative control, 2 µg per IP), H3K9ac antibody (2 µg per IP) on B16 TRCs with various treatments: 5.0 µm WYC‐209: treated with 5.0 µm WYC‐209 for 24 h; LPA + WYC‐209: pretreated with 10 µm LPA for 4 h, then washed out and treated with 5.0 µm WYC‐209 for 20 h; JIB‐04 + WYC‐209: pretreated with 3.0 µm JIB‐04 for 4 h, then washed out and treated with 5.0 µm WYC‐209 for 20 h. (E, F) IP DNA relative to input DNA on P21, P27, P53, *γ*H2AX promoters were determined by qPCR. Mean ± s.e.m.; three independent experiments; one‐way ANOVA testing plus Tukey post‐hoc correction when appropriate was used for statistics. *p < 0.05; **p < 0.01; ***p < 0.001; ns = not significantly different.

To explore how WYC‐209 induces TRC apoptosis, we examined a panel of cell cycle regulatory proteins including P21, P27, P53,^[^ ^20^, ^21^, ^22^ ^]^ and *γ*H2AX.^[^ ^23^, ^24^, ^25^ ^]^ WYC‐209 induced P21, P27, P53, and *γ*H2AX in a time‐ and dose‐dependent manner (Figure S21, Supporting Information). Pretreating TRCs with LPA or Jasp to increase tractions or pretreating with JIB‐04 to increase H3K9me3 inhibited P21, P27, P53, *γ*H2AX, C‐PARP, and C‐Caspase3 elevation by WYC‐209 (**Figure** **6**A–C). Pretreating TRCs with LPA or Jasp inhibited H3K9ac upregulation by WYC‐209 (Figure 6D), demonstrating that LPA or Jasp induced chromatin condensation of TRCs. Pretreating with ML7 or Lat A to inhibit tractions or pretreating with chaetocin to decrease H3K9me3 enhanced P21, P27, P53, *γ*H2AX, C‐PARP, and C‐Caspase 3 expression when compared with WYC‐209 alone (Figure S22A–D, Supporting Information). Taken together, these data suggest that modulating tractions alters chromatin condensation levels and regulates WYC‐209's capacity to change DNA damage protein expressions.

Next, we investigated the underlying mechanism of WYC‐209‐induced chromatin decondensation as well as TRC apoptosis. As chromatin decondensation is a prerequisite for transcriptional activation,^[^ ^26^ ^]^ we deduced that apoptosis‐related genes such as P21, P27, P53 and DNA damage gene *γ*H2AX were altered by chromatin decondensation induced by WYC‐209. ChIP assays show that WYC‐209 increased H3K9ac (Figure 6E) and decreased H3K9me3 (Figure S22E, Supporting Information) at the promoters of P21, P27, P53, and *γ*H2AX in a dose‐dependent manner. Furthermore, increasing F‐actin/tractions via LPA or increasing H3K9me3 via JIB‐04 in TRCs decreased H3K9ac (Figure 6F) and decreasing F‐actin via Lat A or decreasing H3K9me3 via chaetocin in control B16‐F1 increased H3K9ac (Figure S22F, Supporting Information) at the promoters of P21, P27, P53, and *γ*H2AX after WYC‐209 treatment. Together, these results suggest that WYC‐209 induces tumor cell apoptosis via chromatin decondensation to activate apoptosis‐related genes and DNA damage gene expression.

### 2.7 Suppressing H3K9me3 Potentiates WYC‐209 Inhibition of Tumor Metastasis

**Figure 7:**
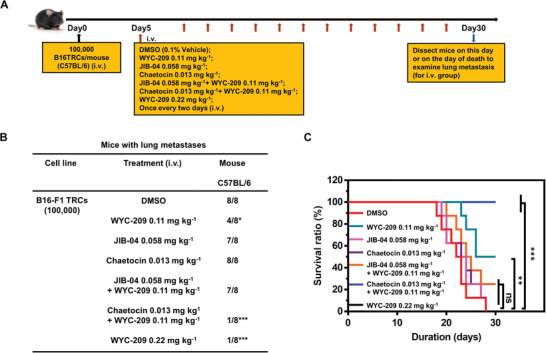
Suppressing H3K9me3 potentiates WYC‐209 inhibition of tumor metastasis. A) Schematic of the experimental protocol; i.v., intravenous injection. Control B16‐F1 cells were cultured in 90 Pa fibrin gels for 5 days to form TRCs. TRCs were isolated from gels and injected intravenously via tail veins into wild‐type immune‐competent syngeneic C57BL/6 mice at 100 000 cells per mouse. Various groups: 8 mice per group were injected with TRCs for 5 days and then were treated with DMSO (0.1%, vehicle) or molecules every 2 days via intravenous injection. Mice were dissected on day 30 or on the day of death to examine signs of lung metastases. B) Lung metastases of B16‐F1 TRCs after being treated with WYC‐209, chaetocin, WYC‐209 with chaetocin, JIB‐04, JIB‐04 with WYC‐209, or DMSO. *p < 0.05; ***p < 0.001 with Fisher's exact test. C) Survival ratio of C57BL/6 mice injected with TRCs treated with different molecules. Statistical analyses of survival ratio were performed by Mantel–Cox test (ns = not significantly different; **p < 0.01; ***p < 0.001 compared to DMSO‐treated group). Note that on day 30 all mice from the DMSO group, chaetocin alone group, and JIB‐04 alone group died; the three lines from three groups overlap, so one only could see the red line. None of the mice from the WYC‐209 0.11 mg kg^−1^ + chaetocin 0.013 mg kg^−1^ group and WYC‐209 0.22 mg kg^−1^ died on day 30; the two lines from two groups overlap, so one only could see the blue line.

To determine whether chromatin decondensation could potentiate WYC‐209 inhibition of tumor metastasis in vivo, we injected intravenously via tail veins 100 000 TRCs into immune‐competent C57BL/6 mice to form lung metastases. Since it has been reported that as few as 10 TRCs are sufficient to generate metastasis in the lung,^[^ ^6^ ^]^ the choice of 100 000 TRCs (10 000‐fold higher than the cell number needed to generate metastasis) is to overload the mice with malignant tumor cells and to determine if modulation of chromatin condensation levels is able to alter the outcome of the retinoid treatment. Five days after loading the mice with TRCs, WYC‐209 was intravenously injected into mice once every two days for 25 days. 0.1% dimethyl sulfoxide (DMSO) was injected as a negative control (**Figure** **7**A). Compared with other groups, none of mice died before 30 days and only one out of 8 mice formed lung metastases when the mice were treated with 0.11 mg kg^−1^ WYC‐209 together with 0.013 mg kg^−1^ chaetocin, similar to the group treated with 0.22 mg kg^−1^ WYC‐209 (Figure 7B,C and Figure S23 (Supporting Information)). By contrast, all mice died before or on day 30 from the DMSO group, the chaetocin (H3K9me3 inhibitor that decondenses the chromatin) alone group, or the JIB‐04 (H3K9me3 demethylase inhibitor) alone group (Figure 7C). Hematoxylin and eosin (H&E) staining of liver, stomach, spleen, bone, and brain shows that no metastases were found in any other organs in various groups (Figure S24, Supporting Information). These results demonstrate that decondensing chromatin with chaetocin substantially potentiates WYC‐209 in inhibiting tumor metastasis and increasing the survival rate of the tumor‐bearing mouse.

## 3 Discussion

Targeted therapy works by aiming at specific genes or proteins that steer cancer growth and survival but few molecules that target cancer metastasis are available. As a synthetic retinoid, WYC‐209 exhibits excellent anticancer activities toward TRCs in various cancer cell lines in vitro and inhibits melanoma TRC metastasis in wild‐type mice with little toxicity.^[^ ^11^ ^]^ Based on the experimental evidence from the current study, we propose a working model of how WYC‐209 induces cancer cell apoptosis. WYC‐209 induces RAR*γ* translocation from the nucleus to the cytoplasm, resulting in a decrease of RAR*γ* binding to the Cdc42 promoter and hence a reduction in Cdc42 expression. Subsequently, low Cdc42 levels lead to F‐actin depolymerization and low contractile forces, which in turn results in chromatin decondensation via decreasing H3K9me3 and increasing H3K9ac, facilitating expression of apoptosis‐related genes and DNA damage gene *γ*H2AX by the retinoid to induce tumor cell apoptosis. Compared with retinoic acid, the endogenous active metabolic compound that regulates embryonic development, cell differentiation, and apoptosis mainly via RAR transcriptional regulations of downstream genes, WYC‐209 is a structurally analog of the third generation retinoid tazarotene.^[^ ^11^ ^]^ It demonstrates selective and efficient inhibition activities toward cancer‐stem‐cell‐like cells TRCs and is more potent than both ATRA and tazarotene.^[^ ^11^ ^]^ Conventional anticancer drugs as ATRA, tazarotene, and cisplatin also induce TRC apoptosis by reducing cell traction and promoting chromatin decondensation. However, conventional drugs are much less potent than WYC‐209 since 10 µm conventional drugs could not induce RAR*γ* translocation as 10 µm WYC‐209 does, which is likely the main reason why WYC‐209 is more effective than conventional anticancer drugs on inducing TRC apoptosis.

S63845 is a small molecule that inhibits antiapoptosis protein myeloid cell leukemia 1 (MCL1) and potently kills MCL1‐dependent cancer cells^[^ ^27^ ^]^ and synergistically (plus Navitoclax) kills difficult‐to‐treat melanoma cells.^[^ ^28^ ^]^ S63845/S64315 (MIK665) are currently under clinical trials (clinical trials.gov; NCT03672695; NCT01989585). Importantly, WYC‐209 was much more potent than S63845 in inhibiting melanoma (B16‐F1) TRCs (Figure S25A,B, Supporting Information). IC_50_ of WYC‐209 was ≈20‐fold lower than that of S63845 (Figure S25C, Supporting Information). Washing out S63845 one day after being treated, colony sizes of TRCs resumed increasing; by contrast, WYC‐209‐treated tumor colony sizes failed to increase after washout (Figure S25D, Supporting Information). The much higher potency of WYC‐209 than S63845 may be partly attributed to different induction mechanisms of apoptosis. In our study, WYC‐209 does not change the total expression levels of RARs (RAR*α*, RAR*β*, and RAR*γ*) in the cells within 24 h, different from the finding of elevation of RAR expressions after retinoic acid treatment.^[^ ^29^, ^30^, ^31^, ^32^ ^]^ However, how WYC‐209 binding to RAR*γ* results in RAR*γ* translocation is not clear at this time and deserves future study. It is possible that RAR*α* and RAR*β* also play roles in WYC‐209‐induced tumor cell apoptosis and elucidation of their specific roles will require additional experiments.

TRCs express less Cdc42 than differentiated tumor cells and Cdc42 regulates TRC stiffness,^[^ ^8^ ^]^ spreading,^[^ ^33^ ^]^ and softness‐dependent extravasation in vivo.^[^ ^34^ ^]^ In addition, TRCs express low focal adhesion kinase (FAK) and downregulate H3K9 methylation through reduction of Cdc42 and RhoA^[^ ^35^ ^]^ that promotes TRC growth via *Sox2*‐dependent self‐renewal.^[^ ^8^ ^]^ Currently, it is unclear how the downregulated Cdc42 and lower tractions by WYC‐209 induces chromatin decondensation. One hypothesis is that the low tractions as a result of low Cdc42 and low F‐actin have direct effects on the chromatin structure to decondense the chromatin. The level of differentiation status of the cancer cells might be a key in controlling endogenous stresses. The results that stem‐cell‐like cells TRCs exhibit lower tractions and lower levels of H3K9me3 and higher levels of H3K9ac than the differentiated melanoma cells (Figure S14, Supporting Information) suggest that these TRCs that have low contractile stresses have decondensed chromatins, consistent with this hypothesis. A report shows that inhibition of myosin‐II‐mediated contractile forces can mitigate large‐nuclear‐deformation‐induced DNA damage,^[^ ^24^ ^]^ suggesting that high contractile stresses may decondense the chromatin structures. The difference in the effect of contractile forces on chromatin condensation between the published report and the current study is not clear at this time but may be partly because the cells are undifferentiated stem‐cell‐like TRCs that are cultured in 3D soft fibrin gels. Alternatively, Cdc42‐mediated F‐actin or traction levels indirectly regulate levels of H3K9ac and H3K9me3. Nevertheless, it will be interesting to elucidate the underlying mechanism in the future.

Gene expression depends on chromatin decondensation to open enough space to allow transcription machinery such as RNA polymerase II binding to gene promoter to initiate gene transcription to complete gene expression followed by complete protein synthesis.^[^ ^36^, ^37^ ^]^ It is logical to enhance apoptosis‐inducing efficiency of WYC‐209 via decreasing cell traction and chromatin condensation. As such, we pretreated or combined F‐actin inhibitor, traction modulators, or chromatin condensation regulators with WYC‐209. These reagents alone did not have effects on cell viability (Figure S19, Supporting Information). Chaetocin alone induced chromatin decondensation but not apoptosis‐related gene expression (Figures S17 and S18, Supporting Information), consistent with the mice experiment data that treating with chaetocin alone does not affect mice survival rate. Hence reducing cellular tension (traction) or decreasing chromatin condensation, combined with WYC‐209, is a safe way to enhance anticancer efficiency of WYC‐209.

It is interesting that 5 µm WYC‐209 abolishes cellular traction to a similar level as 10 µm WYC‐209 but TRC apoptosis induced by 5 µm WYC‐209 is ≈4 times lower than 10 µm WYC‐209 (Figure S1, Supporting Information). Additional mechanisms might be at play here. Expression of DNA repair factor KU80 of TRCs was higher after 5 µm of treatment than after 10 µm treatment (Figure S26A–D, Supporting Information).^[^ ^38^ ^]^ In addition, H3K9me3 was higher and H3K9ac was lower in 5 µm WYC‐209‐treated cells than in 10 µm WYC‐209‐treated cells, suggesting that the chromatin was less decondensed by the 5 µm treatment (Figure S26E,F, Supporting Information). After 24 h treatment by 5 µm WYC‐209, the apoptosis was ≈21%, consistent with Figure S1F,G (Supporting Information), and the apoptosis increased to around 60% after 48 h treatment by 5 µm WYC‐209 (Figure S26G,H, Supporting Information). Together, these data suggest that the additional mechanisms of differences in DNA repair factor KU80 and chromatin decondensation exist for the differences in apoptosis induced by 5 and 10 µm WYC‐209.

In cancer progression, there is increasing evidence that gene expression is governed by epigenetic changes of histones.^[^ ^39^, ^40^ ^]^ Histone deacetylases (HDACs) are enzymes involved in the remodeling of the chromatin, which are aberrant in cancer.^[^ ^41^ ^]^ Several classes of HDAC inhibitors (HDACi) have been found to have potent anticancer activities.^[^ ^42^ ^]^ In our study, WYC‐209 induces melanoma TRC apoptosis via decondensing chromatin and to upregulate apoptotic‐related genes and DNA damage gene, similar to the action of HDACi. However, HDACi monotherapy has been largely ineffective in solid tumors.^[^ ^43^ ^]^ By contrast, WYC‐209 shows a strong inhibition of solid tumor metastasis in vivo at very low concentrations.^[^ ^11^, ^44^ ^]^

Over the last decade or so, a hypothesis that the extracellular matrix stiffens as a result of excessive collagen‐1 is the underlying mechanism of cancer progression has been proposed.^[^ ^45^, ^46^, ^47^ ^]^ A tacit implication of the model is that softening the extracellular matrix by inhibiting collagen‐1 may be a strategy of treating cancer patients. However, a recent study utilizing scaling analysis reveals that there is no correlation between collagen‐1 and survival time of patients for more than two dozens of solid tumors;^[^ ^48^ ^]^ importantly, for the only type of cancer (liver cancer) that is associated with collagen‐1, the finding is the opposite: more collagen‐1 and stiffer extracellular matrix are associated with longer survival time of patients. In fact, decreasing matrix content and lowering tissue stiffness in pancreatic ductal adenocarcinoma mouse models lead to accelerated tumor growth and diminished overall survival.^[^ ^49^ ^]^ Together with the fact that corticosteroids that are often used to alleviate cancer patients of pain and inflammation can inhibit collagen‐1 synthesis but cannot inhibit cancer growth or progression and are potentially a risk factor for failure of solid tumor treatment,^[^ ^50^ ^]^ it supports a postulate that excessive collagen‐1 and the stiffened matrix are a protective response of the body to fight against tumor size expansion. This protective mechanism fails in some cases as tumor cells can break down the stiffened matrix by secreting matrix‐degrading enzymes and invade nearby tissues to initiate tumor progression. The finding that a stiffened extracellular matrix in fact induces dormancy of malignant TRCs in mice^[^ ^7^ ^]^ is consistent with this interpretation. Differentiated tumor cells are stiff, less malignant, and less metastatic than the soft TRCs and stiffening and differentiating TRCs by retinoic acid can inhibit TRC growth.^[^ ^6^, ^8^ ^]^ By contrast, in this study, we demonstrate that while softening the TRCs and abrogating tractions induce chromatin decondensation of TRCs, they cannot kill the TRCs but can potentiate the efficacy of the synthetic retinoid in inducing TRC apoptosis to inhibit cancer progression.

TRCs are cancer‐stem‐cell‐like cells which are resistant to conventional anticancer drugs such as cisplatin, ATRA, and tazarotene. It is remarkable that WYC‐209 is not only much more potent than these anticancer drugs, but also more potent on TRCs than differentiated tumor cells (Figure S27, Supporting Information). TRCs are soft with decondensed chromatin and WYC‐209 induces cell apoptosis accompanied with cellular tension reduction and chromatin decondensation, suggesting that decreasing cellular tension or inducing chromatin decondensation could be an effective strategy for treating TRCs or cancer‐stem‐cell‐like cells.

In summary, we show that the retinoid WYC‐209 and other conventional anticancer drugs as ATRA, tazarotene, and cisplatin induce melanoma TRC apoptosis via eliminating tractions to decondense chromatin and upregulate apoptotic‐related genes and DNA damage gene. Combining histone demethylation molecule chaetocin with WYC‐209 enhances inhibitory capacity of the synthetic retinoid in melanoma metastasis to the lung. Our findings unveil a new mechanism which lowers tension and decondenses chromatin to effectively enhance TRC apoptosis and DNA damage by anticancer drugs. We propose a therapeutic strategy of enhancing cancer inhibition via abolishing cell tension and inducing chromatin decondensation. In the future, it will be interesting to determine if this combined approach could inhibit growth and metastasis of various types of cancer cells in animal models and in patients.

## 4 Experimental Section

### Animals

Four‐week old female C57BL/6 mice were purchased from the Center of Medical Experimental Animals of Hubei Province (Wuhan, China). The mice were randomly assigned to all experiment groups. All animals received human care in compliance with the Principles of Laboratory Animal Care Formulated by the National Society of Medical Research and the guide for the US National Institutes of Health. The protocol was approved by the Institutional Animal Care and Use Committee (IACUC) of Huazhong University of Science and Technology. The animal studies performed were IACUC approved. The IACUC number was 2596.

### Cell Culture

Murine melanoma cell line B16‐F1 was obtained from the China Center for Type Culture Collection (CCTCC, Wuhan, China). B16‐F1 was cultured in Roswell Park Memorial Institute (RPMI)‐1640 (Hyclone, South Logan, UT, USA), supplemented with 10% fetal bovine serum (Invitrogen) and 1% penicillin–streptomycin (Sigma, St. Louis, MO, USA) at 37 °C with 5% CO_2_. The cells were randomly assigned to each dish and DNA 4',6‐diamidino‐2‐phenylindole (DAPI) staining was constantly monitored to see if there were signs of mycoplasma contamination and no contamination was observed.

### Synthesis of the Retinoid WYC‐209

The synthesis work for WYC‐209 followed the reported procedure.^[^ ^11^ ^]^ Briefly, a Sonogashira coupling was adopted for the connection of 6‐ethynyl‐4, 4‐dimethylthiochroman and ethyl 2‐chloropyrimidine‐5‐carboxylate moieties under the Pd(PPh_3_)_2_Cl_2_–CuI catalytic condition. The coupling intermediate was oxidized to sulfoxide product under *m*‐chloroperbenzoic acid (*m*‐CPBA) condition. WYC‐209 was a 1:1 mixture of WYC‐209A and WYC‐209B, both being active molecules in inhibiting cancer cells in vitro with similar IC_50_.^[^ ^11^ ^]^

### 3D Fibrin Gel Preparation

TRC culture method was described previously.^[^ ^6^ ^]^ Briefly, the same volume of fibrinogen (Reagent Proteins, San Diego, CA, USA) and single‐cell solution mixture were seeded into each well of a 24‐well plate, resulting in 1 mg mL^−1^ fibrin gels (≈90 Pa) and 6000 cells in the 245 µL mixture, mixed well with 5 µL preadded thrombin (100 U mL^−1^, Reagent Proteins). The cell culture plate was then incubated in 37 °C cell culture incubator for 30 min. Finally, 1 mL of RPMI‐1640 or Dulbecco's modified Eagle medium containing 10% fetal bovine serum and antibiotics was added.

### TUNEL Assay

TUNEL Apoptosis Detection kits (Abbkine, CA, USA, KTA2011) and DAPI (Biosharp, BS097) were used to label apoptotic cells and cell nucleus. Briefly, controls and TRCs treated with different conditions were fixed with 4% paraformaldehyde for 30 min at room temperature, and permeabilized with 0.2% Triton X‐100 in phosphate‐buffered saline (PBS) for 10 min. TUNEL reaction was performed. After TUNEL labeling, nucleus was labeled with DAPI in blue and the TUNEL‐positive‐labeled cells were in red, which were observed by DMI‐6000B fluorescence microscope.

### Flow Cytometry

For Annexin‐V and propidium iodide (PI) staining to quantify cell apoptosis, treated B16 Ctrs and B16 TRCs were labeled with fluorescein isothiocyanate (FITC)–Annexin‐V and PI according to the manufacturer's instruction (BD Pharmingen, San Diego, CA, USA, 556547). Data were acquired on a cytoFLEX (Beckman Coulter) and analyzed with CytExpert software.

### IC_50_ Assay

The IC_50_ was measured by MTT colorimetric assay (Abcam, Cambridge, MA, USA, ab211091). Controls or TRCs seeded in 96‐well microplates at a density to maintain control (untreated) cells in an exponential phase of growth during the entire experiment. Cells were incubated with various treatment conditions. For each incubation, 50 µL MTT reagent (Abcam) was well added for 3 h at 37 °C. After incubation, 150 µL MTT solvent (Abcam) was added and absorbance was measured at 570 nm. All experiments were repeated at least 3 times. Cell growth inhibition = (1 − optical density (OD) treated cells/OD control cells) × 100%. The IC_50_ values were determined with XLFit or Graphpad Prism curve fitting software. Cisplatin (HY‐17394), tazarotene (HY‐15388), and ATRA (HY‐14649) were obtained from MedChem Express (NJ, USA). S63845 (S8383) was purchased from Selleck.

### Small Interfering RNAs and Reagents

Cells were transfected with siRNA using Lipofectamine 3000 (Invitrogen, L3000015) following the manufacturer's protocol. The sequences of siRNAs were: scrambled siRNA: 5ʹ‐UUCUCCGAACGUGUCACGUTT‐3ʹ (sense) and 5ʹ‐ACGUGACACGUUCGGAGAATT‐3ʹ (antisense); mouse Cdc42 siRNA #1: 5ʹ‐GGGCAAGAGGAUUAUGACATT‐3ʹ (sense) and 5ʹ‐UGUCAUAAUCCUCUUGCCCTT‐3ʹ (antisense); mouse Cdc42 siRNA #2: 5ʹ‐ AGAAGUUAGAA‐AUUAUUCCCC‐3ʹ (sense) and 5ʹ‐GGAAUAAUUUCUAACUUCUUU‐3ʹ (antisense); mouse SUV39h1 siRNA#1: 5ʹ‐GGUCCUUUGUCUAUAUCAATT‐3ʹ (sense) and 5ʹ‐ UUGAUAUAGAC‐AAAGGACCTT‐3ʹ (antisense); mouse SUV39h1 siRNA#2: 5ʹ‐ GGUGUACAACGUAUUCAUATT‐3ʹ (sense) and 5ʹ‐UAUGAAUACGUUGUACACCTT‐3ʹ (antisense); mouse RAR*α*: 5ʹ‐UAAAUGUGC‐UUAAAAUGAATT‐3ʹ (sense) and 5ʹ‐UUCAUUUUAAGCACAUUUAUA‐3ʹ (antisense); mouse RAR*β*: 5ʹ‐UAUCUGGGGAUUGGUACGCTT‐3ʹ (sense) and 5ʹ‐GCGUACCAAUCCCCAGAUATT‐3ʹ (antisense); mouse RAR*γ*: 5ʹ‐CUAAUAAAUAAAUAGAGGCTT‐3ʹ (sense) and 5ʹ‐GCCUCUAUU‐UAUUUAUUAGCU‐3ʹ (antisense). Jasplakinolide (sc‐202191) and MM11253 (sc‐361255) were obtained from Santa Cruz (CA, USA). JIB‐04 (HY‐13953), chaetocin (HY‐N2019), lysophosphatidic acid (HY‐137862), ML‐7 (HY‐15417), trichostatin A (HY‐15144), and latrunculin A (HY‐16929) were obtained from MedChem Express (NJ, USA).

### Plasmid Transfection

B16‐F1 was transfected with complementary DNA (cDNA) using Lipofectamine 3000 (Invitrogen) following the manufacturer's protocol. plasmid cloning DNA (pcDNA)3.1–Cdc42 plasmid or pcDNA3.1–GFP (Emp Vec) were purchased from GenePharma (Shanghai, China).

### 3D Traction Quantification

The method of ERMGs to measure 3D cell traction in a 3D cell colony was described previously.^[^ ^13^ ^]^ Briefly, 20 µL cell suspension (5 × 10^3^ cells), 5 µL ERMG solution (stock concentration of EGRM was 6 × 10^7^ ERMGs mL^−1^), and 25 µL fibrinogen solution (2 mg mL^−1^) were mixed into a 35 mm glass‐bottomed dish preadded with 1 µL thrombin (100 U mL^−1^), resulting in 90 Pa soft fibrin gels culturing for 3 days. After drug treatment, z‐stacks were acquired with 0.3 µm step and 512 × 512 focal planes with a pixel size of ≈0.15 µm over a 45–60 s period with line averaging of 3. After acquiring 3D stacks of stressed images, the images were acquired again after treating with 10% Triton X‐100 solution (Sigma, 93443) for ≈0.5 h until the microgel was no longer stressed. Then, traction calculation was based on these 3D confocal images in MATLAB. All images were acquired with a Leica SP8 (Wetzlar, Germany) confocal microscope.

### Western Blot Analyses and Antibodies

The cell lysates were prepared with radio immunoprecipitation assay (RIPA) lysis buffer (Beyotime, Jiangsu, China, P0013B) containing proteinase inhibitors (Roche, 04693132001). The protein concentration was quantified by the bicinchoninic acid (BCA) kit (Beyotime, P0010) according to the manufacturer's instructions. Samples were separated by a sodium dodecyl sulfate‐polyacrylamide gel electrophoresis (SDS‐PAGE) gel and transferred to a nitrocellulose membrane (GE Healthcare), and then blocked with 5% bovine serum albumin (BSA, Sigma, SRE0098). All primary antibodies were diluted in blocking solution and incubated overnight at 4 °C: Caspase 3 (CST, Danvers, MA, USA, 1:1000, #9662), cleaved Caspase 3 (CST, 1:1000, #9664), cleaved PARP (CST, 1:1000, #9548), P21 (Santa Cruz, 1:1000, sc‐6246), P27 (CST, 1:1000, #3686), P53 (CST, 1:1000, #2524), H3K9me3 (CST, 1:1000, #13969), H3K9ac (CST, 1:1000, #9649), RAR alpha (CST, 1:1000, #62294), RAR beta (Abcam, 1:1000, ab124701), RAR gamma (Abcam, 1:1000, ab191368), p‐MLC2 (CST, 1:1000, #3675), Cdc42 (CST, 1:1000, #2466), gamma H2AX (Abcam, 1:1000, ab 26350), SUV39h1 (CST, 1:1000, #8729), glyceraldehyde‐3‐phosphate dehydrogenase (GAPDH) (Abcam, 1:1000, ab8245), and beta‐tubulin (Proteintech, Wuhan, China, 1:1000, 10094‐1‐AP). Primary antibodies were detected with anti‐mouse immunoglobulin G (IgG) (H+L) (DyLight 800 4X PEG Conjugate) (CST, 1:20 000, #5257) or anti‐rabbit IgG (H+L) (DyLight 800 4X PEG Conjugate) (CST, 1:20 000, #5151).

### Cell Fractionation

After treatment, the cell cytoplasmic and nuclear proteins were isolated by using the Cell Fractionation kit (Abcam, ab109719) according to the supplier's instruction. Equal cell equivalents were run on SDS‐PAGE gels and immunoblotted.

### Immunofluorescence

Cells were fixed with 4% formaldehyde (BioSharp, BL539A) for 30 min at room temperature. Then, cells were permeabilized with 0.5% Triton X‐100 for 5 min. Fixed cells were blocked in 5% BSA for 1 h at room temperature and probed with H3K9me3 (CST, 1:1000, #13969), H3K9ac (CST, 1:400, #9649), RAR gamma (Abcam, 1:1000, ab191368), and KU80 (Abcam, 1:1000, ab80592) antibodies in blocking solution overnight at 4 °C. Then, cells were washed in PBS and incubated with Alexa‐Fluor‐594‐conjugated secondary antibody (Abcam, 1:1000, ab150080) and DAPI (Biosharp, BS097) for 2 h under dark conditions. Images were acquired with a Leica SP8 (Wetzlar, Germany, 63× objective at 1024 × 1024 resolution with 2 times zoom) confocal microscope.

### Chromatin Immunoprecipitation Assay

ChIP assays were performed following the manufacturer's instructions (Simple ChIP Enzymatic Chromatin IP kit, CST, #9005). Briefly, cells were subjected to cross‐linking with 1% formaldehyde in medium for 10 min at 37 °C followed by glycine treatment for 5 min that quenched the formaldehyde. Chromatin was sonicated to shear DNA to an average length of 0.2–1.0 kb. ChIP was performed using a control rabbit IgG or antibody against H3K9me3 (CST, #13969), H3K9ac (CST, #9649), or RAR*γ* (Proteintech, 11424‐1‐AP). The DNA fragments in the precipitates were purified for real‐time quantitative polymerase chain reaction (qPCR) analysis. For ChIP–qPCR experiments, qPCR was conducted using SYBR Green Supermix (Bio‐Rad, Hercules, CA, USA, #1725271) on CFX Connect Real‐Time PCR Detection System (Bio‐Rad). qPCR primers were designed and manufactured by Sigma. The primer sequences were listed below as follows: mouse P21 promoter primer set, GATCGGTGA‐AGGAGTGGGTT (forward) and TGTCTGGATATCGCTGTGGATC (reverse); mouse P27 promoter primer set, AGACCAATGGAGCTCCTCCT (forward) and TGGCAAACAGTCGG‐AGCGTA (reverse); mouse P53 promoter primer set, TGGGATTGGGACTTTCCCCT (forward) and GGTCTCGTCACGCTCATCAA (reverse); mouse H2AX promoter primer set, GAACCAATCAGGAGGAAGCG(forward) and CCGGACATAGTGTACGAGGTAG (reverse); mouse Cdc42 promoter primer set, AGATCAACCTCTGGTACCTAGT (forward) and GCTAGCCTGGAACTCAGAGAA (reverse).

### Real‐Time (RT) Analysis

Total mRNA was isolated from B16 cells using TRIzol reagent according to the supplier's instruction (Invitrogen, 15596018). Expression changes of genes were performed using HiScript II One Step quantitative real‐time polymerase chain reaction (qRT‐PCR) SYBR Green Kit (Vazyme, Q221‐01) with a reaction condition of reverse transcription at 50 °C for 15 min, initial denaturation at 95 °C for 5 min, and then 40 cycles of 95 °C for 10 s and 60 °C for 30 s on an CFX Connect Real‐Time System (Bio‐Rad, Hercules, CA, USA). The data were normalized against mouse GAPDH. The primer sequences were as follows: mouse GAPDH forward primer: 5′‐AGGTCGGTGTGAACGGATTTG‐3′ and reverse primer: 5′‐ TGTAGACCATGTAGTT‐GAGGTCA‐3′; mouse RAR*α* forward primer: 5′‐TCAGTGCCATCTGCCTCATCT‐3′ and reverse primer: 5′‐ATGCTCCGAAGGTCTGTGATCT‐3′; mouse RAR*β* forward primer: 5′‐CTGCTTGCCTGGACATCCTAAT‐3′ and reverse primer: 5′‐CAGTCTCGGTGTCATCC‐ATCTC‐3′; mouse RAR*γ* forward primer: 5′‐AATGCTGGCTTCGGTCCTCT‐3′ and reverse primer:5′‐ CCTGGCGGTCTCCACAGATTA‐3′.

### Luciferase Reporter Assay

The plasmids for RAR*γ* overexpression (pcDNA3.1–GFP–RAR*γ*), RAR*γ* NC (pcDNA3.1–GFP–RAR*γ* NC), and promoter reporter Cdc42 (GPL3‐Basic‐Cdc42), Cdc42 NC (GPL3‐Basic‐Cdc42 NC), and TK (pRL‐TK) were constructed by GenePharma Corporation (Suzhou, China). Plasmids were transiently transfected into B16 TRCs. After 48 h culture, B16 TRCs treated with 10 µm WYC‐209 for 1 h or not, then the luciferase activities were measured by a Dual Luciferase Reporter Gene Assay Kit according to manufacturer's instructions (RG027, Beyotime, China).

### Chromatin Electron Microscopy Image

Collecting cell precipitation after various treatments, then the precipitation in 0.2% glutaraldehyde solution at 4 °C was fixed. The fixed cells were washed by PBS for 3 times and wrapped into agarose. Agarose blocks with samples were post fixed with 1% OsO4 in PBS for 2 h, then dehydrated, resin penetration and embedding, polymerization, ultrathin section, staining, and images captured under transmission electron microscope (HITACHI, HT7800/HT7700).

### Mice Experiments

Four‐week old female C57BL/6 mice were used in mice experiment. Mice were randomized into different groups. TRCs (B16‐F1) were selected from 3D 90 Pa fibrin gels for 5 days and pipetted to single cells in PBS with appropriate cell density (1 × 10^6^ mL^−1^). 100 µL single cell solution (100 000 TRCs) was intravenously injected into the tail vein of each wild‐type C57BL/6 mouse. Five days later, inoculated mice were intravenous implanted with DMSO (0.1%); JIB‐04 (0.058 mg kg^−1^); chaetocin (0.013 mg kg^−1^); WYC‐209 (0.11 mg kg^−1^); WYC‐209 (0.22 mg kg^−1^); JIB‐04 (0.058 mg kg^−1^) with WYC‐209 (0.11 mg kg^−1^) or chaetocin (0.013 mg kg^−1^) with WYC‐209 (0.11 mg kg^−1^) every two days. The mice were euthanized and examined for lung tumor formation at day 30 or at the day of death.

### Histologic Evaluation and Immunohistochemistry

Lung, liver, spleen, bone, stomach, and brain of C57BL/6 mice were fixed by 4% paraformaldehyde, then were embedded in paraffin and cut to ≈4 µm thick sections by Thermo FINESSE 325. Organ sections were stained by H&E and slides were evaluated for tumor formation by a veterinary pathologist blinded to sample identity.

### Statistical Analysis

Graphpad Prism software was used for generating Kaplan–Meier animal survival plots of 0.1% DMSO and compound‐treated mice and performing statistical analysis (using a log‐rank test (Mantel–Cox)). Mice lung metastasis experiments were analyzed by Fisher's exact test. All other experimental data were analyzed using a two‐tailed Student's *t*‐test and one‐way analysis of variance (ANOVA) test, when multiple comparisons were carried out within a set of experiments, Tukey post‐hoc correction was also performed. Graphpad Prism was used to generate IC_50_ curves treated with WYC‐209 and other compounds in vitro. All data were expressed as mean ±  standard error of the mean (s.e.m.) and significance was defined as *p* ≤ 0.05. Statistical analysis was carried out using Graphpad Prism software.

## Conflict of Interest

The authors declare no conflict of interest.

## Author Contributions

N.W. and J.C. conceived the project. Y.Z., X.C., N.W., and J.C. designed the experiments. Y.Z., Q.D., Q.A., C.Z., E.M., B.N., F.Q., F.W., S.C., X.C., and A.W. performed experiments and analyses. Y.Z., X.C., N.W., and J.C. wrote the paper with inputs from other authors.

## Supporting information

Supporting Information

## Data Availability

The data that support the findings of this study are available in the supplementary material of this article.

## References

[1] P. L. Bedard , D. M. Hyman , M. S. Davids , L. L. Siu , Lancet 2020, 395, 1078. 32222192 10.1016/S0140-6736(20)30164-1

[2] C. Holohan , S. Van Schaeybroeck , D. B. Longley , P. G. Johnston , Nat. Rev. Cancer 2013, 13, 714. 24060863 10.1038/nrc3599

[3] J. E. Visvader , G. J. Lindeman , Cell Stem Cell 2012, 10, 717. 22704512 10.1016/j.stem.2012.05.007

[4] T. N. Almanaa , M. E. Geusz , R. J. Jamasbi , J. Cancer 2013, 4, 536. 23983818 10.7150/jca.6477 PMC3753528

[5] M. Dean , T. Fojo , S. Bates , Nat. Rev. Cancer 2005, 5, 275. 15803154 10.1038/nrc1590

[6] J. Liu , Y. Tan , H. Zhang , Y. Zhang , P. Xu , J. Chen , Y.‐C. Poh , K. Tang , N. Wang , B. Huang , Nat. Mater. 2012, 11, 734. 22751180 10.1038/nmat3361 PMC3405191

[7] Y. Liu , J. Lv , X. Liang , X. Yin , L. Zhang , D. Chen , X. Jin , R. Fiskesund , K. Tang , J. Ma , H. Zhang , W. Dong , S. Mo , T. Zhang , F. Cheng , Y. Zhou , J. Xie , N. Wang , B. Huang , Cancer Res. 2018, 78, 3926. 29764867 10.1158/0008-5472.CAN-17-3719

[8] Y. Tan , A. Tajik , J. Chen , Q. Jia , F. Chowdhury , L. Wang , J. Chen , S. Zhang , Y. Hong , H. Yi , D. C. Wu , Y. Zhang , F. Wei , Y.‐C. Poh , J. Seong , R. Singh , L.‐J. Lin , S. Doğanay , Y. Li , H. Jia , T. Ha , Y. Wang , B. Huang , N. Wang , Nat. Commun. 2014, 5, 4619. 25099074 10.1038/ncomms5619 PMC4133791

[9] X. H. Tang , L. J. Gudas , Annu. Rev. Pathol. 2011, 6, 345. 21073338 10.1146/annurev-pathol-011110-130303

[10] D. Wang , S. J. Lippard , Nat. Rev. Drug Discovery 2005, 4, 307. 15789122 10.1038/nrd1691

[11] J. Chen , X. Cao , Q. An , Y. Zhang , K. Li , W. Yao , F. Shi , Y. Pan , Q. Jia , W. Zhou , F. Yang , F. Wei , N. Wang , B. Yu , Nat. Commun. 2018, 9, 1406. 29643385 10.1038/s41467-018-03877-7 PMC5895803

[12] H. T. Nia , L. L. Munn , R. K. Jain , Science 2020, 370, eaaz0868. 33122355 10.1126/science.aaz0868 PMC8274378

[13] E. Mohagheghian , J. Luo , J. Chen , G. Chaudhary , J. Chen , J. Sun , R. H. Ewoldt , N. Wang , Nat. Commun. 2018, 9, 1878. 29760452 10.1038/s41467-018-04245-1 PMC5951850

[14] X. Zhao , C. Graves , S. J. Ames , D. E. Fisher , R. A. Spanjaard , Cancer Res. 2009, 69, 5218. 19470764 10.1158/0008-5472.CAN-09-0705

[15] G. Brown , K. Petrie , Int. J. Mol. Sci. 2021, 22, 3632. 33807298 10.3390/ijms22073632 PMC8036636

[16] S.‐J. Heo , K. H. Song , S. Thakur , L. M. Miller , X. Cao , A. P. Peredo , B. N. Seiber , F. Qu , T. P. Driscoll , V. B. Shenoy , M. Lakadamyali , J. A. Burdick , R. L. Mauck , Sci. Adv. 2020, 6, eaax5083. 32596438 10.1126/sciadv.aax5083 PMC7304973

[17] K. Damodaran , S. Venkatachalapathy , F. Alisafaei , A. V. Radhakrishnan , D. S. Jokhun , V. B. Shenoy , G. V. Shivashankar , Mol. Biol. Cell 2018, 29, 3039. 30256731 10.1091/mbc.E18-04-0256 PMC6333178

[18] L. Wang , J. Chang , D. Varghese , M. Dellinger , S. Kumar , A. M. Best , J. Ruiz , R. Bruick , S. Peña‐Llopis , J. Xu , D. J. Babinski , D. E. Frantz , R. A. Brekken , A. M. Quinn , A. Simeonov , J. Easmon , E. D. Martinez , Nat. Commun. 2013, 4, 2035. 23792809 10.1038/ncomms3035 PMC3724450

[19] D. Greiner , T. Bonaldi , R. Eskeland , E. Roemer , A. Imhof , Nat. Chem. Biol. 2005, 1, 143. 16408017 10.1038/nchembio721

[20] Y. Xiong , G. J. Hannon , H. Zhang , D. Casso , R. Kobayashi , D. Beach , Nature 1993, 366, 701. 8259214 10.1038/366701a0

[21] A. C. Carrano , E. Eytan , A. Hershko , M. Pagano , Nat. Cell Biol. 1999, 1, 193. 10559916 10.1038/12013

[22] H. Hong , K. Takahashi , T. Ichisaka , T. Aoi , O. Kanagawa , M. Nakagawa , K. Okita , S. Yamanaka , Nature 2009, 460, 1132. 19668191 10.1038/nature08235 PMC2917235

[23] L. J. Mah , A. El‐Osta , T. C. Karagiannis , Leukemia 2010, 24, 679. 20130602 10.1038/leu.2010.6

[24] Y. Xia , C. R. Pfeifer , K. Zhu , J. Irianto , D. Liu , K. Pannell , E. J. Chen , L. J. Dooling , M. P. Tobin , M. Wang , I. L. Ivanovska , L. R. Smith , R. A. Greenberg , D. E. Discher , J. Cell Biol. 2019, 218, 2545. 31239284 10.1083/jcb.201811100 PMC6683732

[25] C. R. Pfeifer , Y. Xia , K. Zhu , D. Liu , J. Irianto , V. M. M. García , L. M. S. Millán , B. Niese , S. Harding , D. Deviri , R. A. Greenberg , D. E. Discher , Mol. Biol. Cell 2018, 29, 1948. 29742017 10.1091/mbc.E18-02-0079 PMC6232975

[26] P. Therizols , R. S. Illingworth , C. Courilleau , S. Boyle , A. J. Wood , W. A. Bickmore , Science 2014, 346, 1238. 25477464 10.1126/science.1259587 PMC6529354

[27] A. Kotschy , Z. Szlavik , J. Murray , J. Davidson , A. L. Maragno , G. L. Toumelin‐Braizat , M. Chanrion , G. L. Kelly , J.‐N. Gong , D. M. Moujalled , A. Bruno , M. Csekei , A. Paczal , Z. B. Szabo , S. Sipos , G. Radics , A. Proszenyak , B. Balint , L. Ondi , G. Blasko , A. Robertson , A. Surgenor , P. Dokurno , I. Chen , N. Matassova , J. Smith , C. Pedder , C. Graham , A. Studeny , G. Lysiak‐Auvity , et al., Nature 2016, 538, 477. 27760111 10.1038/nature19830

[28] N. Mukherjee , J. Skees , K. J. Todd , D. A. West , K. A. Lambert , W. A. Robinson , C. M. Amato , K. L. Couts , R. Van Gulick , M. MacBeth , K. Nassar , A.‐C. Tan , Z. Zhai , M. Fujita , S. M. Bagby , C. R. Dart , J. R. Lambert , D. A. Norris , Y. G. Shellman , Cell Death Dis. 2020, 11, 443. 32513939 10.1038/s41419-020-2646-2 PMC7280535

[29] J. W. Zhang , J. Y. Wang , S. J. Chen , Z. Chen , J. Biosci. 2000, 25, 275. 11022230 10.1007/BF02703936

[30] Y. Shang , C. R. Baumrucker , M. H. Green , J. Biol. Chem. 1999, 274, 18005. 10364250 10.1074/jbc.274.25.18005

[31] C.‐S. Koh , J.‐L. Ku , S.‐Y. Park , K.‐H. Kim , J.‐S. Choi , I.‐J. Kim , J.‐H. Park , S. K. Oh , J.‐K. Chung , J.‐H. Lee , W. H. Kim , C. W. Kim , B. Y. Cho , J.‐G. Park , Mol. Cell. Endocrinol. 2007, 264, 118. 17134824 10.1016/j.mce.2006.10.017

[32] C. Wang , D. Zhao , K. Wang , L. Gao , Y. He , H. Wu , L. Ruan , W. Chen , D. Zhang , T. Xia , S. Qian , Z. Liu , Y. Yang , W. Yang , A. Hu , Q. Zhao , Nutr. Cancer 2021, 73, 2065. 32959699 10.1080/01635581.2020.1823006

[33] F. Chowdhury , S. Doğanay , B. J. Leslie , R. Singh , K. Amar , B. Talluri , S. Park , N. Wang , T. Ha , Biochem. Biophys. Res. Commun. 2018, 500, 557. 29673588 10.1016/j.bbrc.2018.04.085 PMC6133653

[34] J. Chen , W. Zhou , Q. Jia , J. Chen , S. Zhang , W. Yao , F. Wei , Y. Zhang , F. Yang , W. Huang , Y. Zhang , H. Zhang , Y. Zhang , B. Huang , Z. Zhang , H. Jia , N. Wang , Sci. Rep. 2016, 6, 19304. 26787224 10.1038/srep19304 PMC4726408

[35] Y. Tan , A. R. Wood , Q. Jia , W. Zhou , J. Luo , F. Yang , J. Chen , J. Chen , J. Sun , J. Seong , A. Tajik , R. Singh , N. Wang , Biochem. Biophys. Res. Commun. 2017, 483, 456. 28007596 10.1016/j.bbrc.2016.12.122 PMC5253317

[36] I. Jonkers , J. T. Lis , Nat. Rev. Mol. Cell Biol. 2015, 16, 167. 25693130 10.1038/nrm3953 PMC4782187

[37] J. Sun , J. Chen , E. Mohagheghian , N. Wang , Sci. Adv. 2020, 6, eaay9095. 32270037 10.1126/sciadv.aay9095 PMC7112933

[38] J. Irianto , Y. Xia , C. R. Pfeifer , A. Athirasala , J. Ji , C. Alvey , M. Tewari , R. R. Bennett , S. M. Harding , A. J. Liu , R. A. Greenberg , D. E. Discher , Curr. Biol. 2017, 27, 210. 27989676 10.1016/j.cub.2016.11.049 PMC5262636

[39] A. H. Lund , M. van Lohuizen , Genes Dev. 2004, 18, 2315. 15466484 10.1101/gad.1232504

[40] S. B. Baylin , J. E. Ohm , Nat. Rev. Cancer 2006, 6, 107. 16491070 10.1038/nrc1799

[41] A. M. Valencia , C. Kadoch , Nat. Cell Biol. 2019, 21, 152. 30602726 10.1038/s41556-018-0258-1 PMC6755910

[42] P. A. Marks , X. Jiang , Cell Cycle 2005, 4, 549. 15738652 10.4161/cc.4.4.1564

[43] Y. Li , E. Seto , Cold Spring Harbor Perspect. Med. 2016, 6, a026831. 10.1101/cshperspect.a026831 PMC5046688 27599530

[44] F. Qi , W. Qin , Y. Zhang , Y. Luo , B. Niu , Q. An , B. Yang , K. Shi , Z. Yu , J. Chen , X. Cao , J. Xia , J. Exp. Clin. Cancer Res. 2021, 40, 280. 34479623 10.1186/s13046-021-02085-4 PMC8418008

[45] M. J. Paszek , N. Zahir , K. R. Johnson , J. N. Lakins , G. I. Rozenberg , A. Gefen , C. A. Reinhart‐King , S. S. Margulies , M. Dembo , D. Boettiger , D. A. Hammer , V. M. Weaver , Cancer Cell 2005, 8, 241. 16169468 10.1016/j.ccr.2005.08.010

[46] D. T. Butcher , T. Alliston , V. M. Weaver , Nat. Rev. Cancer 2009, 9, 108. 19165226 10.1038/nrc2544 PMC2649117

[47] O. Maller , A. P. Drain , A. S. Barrett , S. Borgquist , B. Ruffell , I. Zakharevich , T. T. Pham , T. Gruosso , H. Kuasne , J. N. Lakins , I. Acerbi , J. M. Barnes , T. Nemkov , A. Chauhan , J. Gruenberg , A. Nasir , O. Bjarnadottir , Z. Werb , P. Kabos , Y.‐Y. Chen , E. S. Hwang , M. Park , L. M. Coussens , A. C. Nelson , K. C. Hansen , V. M. Weaver , Nat. Mater. 2021, 20, 548. 33257795 10.1038/s41563-020-00849-5 PMC8005404

[48] M. Vashisth , S. Cho , J. Irianto , Y. Xia , M. Wang , B. Hayes , D. Wieland , R. Wells , F. Jafarpour , A. Liu , D. E. Discher , Proc. Natl. Acad. Sci. USA 2021, 118, e2112940118. 34810266 10.1073/pnas.2112940118 PMC8640833

[49] H. Jiang , R. J. Torphy , K. Steiger , H. Hongo , A. J. Ritchie , M. Kriegsmann , D. Horst , S. E. Umetsu , N. M. Joseph , K. McGregor , M. J. Pishvaian , E. M. Blais , B. Lu , M. Li , M. Hollingsworth , C. Stashko , K. Volmar , J. J. Yeh , V. M. Weaver , Z. J. Wang , M. A. Tempero , W. Weichert , E. A. Collisson , J. Clin. Invest. 2020, 130, 4704. 32749238 10.1172/JCI136760 PMC7456216

[50] H. P. Rutz , Lancet 2002, 360, 1969. 12493280 10.1016/S0140-6736(02)11922-2

