# Supplementary material for: Synthetic Retinoid Kills Drug‐Resistant Cancer Stem Cells via Inducing RARγ‐Translocation‐Mediated Tension Reduction and Chromatin Decondensation
Source: Adv Sci (Weinh). 2022 Aug 28;9(31):2203173. doi: 10.1002/advs.202203173 (PMC9631059; doi:10.1002/advs.202203173)
Supplement: Supplementary file 1 — Supporting Information [file ADVS-9-2203173-s001.pdf]

ADVANCED SCIENCE
Open Access

Supporting Information

for *Adv. Sci.*, DOI 10.1002/advs.202203173

Synthetic Retinoid Kills Drug-Resistant Cancer Stem Cells via Inducing RARγ-Translocation-Mediated Tension Reduction and Chromatin Decondensation

Yao Zhang, Qi Dong, Quanlin An, Chumei Zhang, Erfan Mohagheghian, Bing Niu, Feng Qi, Fuxiang Wei, Sihan Chen, Xinman Chen, Anqi Wang, Xin Cao, Ning Wang* and Junwei Chen**

Supporting Information

Synthetic retinoid kills drug-resistant cancer stem cells via inducing RARγ translocation mediated tension reduction and chromatin decondensation

Yao Zhang, Qi Dong, Quanlin An, Chumei Zhang, Erfan Mohagheghian, Bing Niu, Feng Qi, Fuxiang Wei, Sihan Chen, Xinman Chen, Anqi Wang, Xin Cao, Ning Wang*, and Junwei Chen**

*Send correspondence to:

Dr. Junwei Chen, chenjunwei@hust.edu.cn

Dr. Ning Wang, nwangrw@illinois.edu

Dr. Xin Cao, caox@fudan.edu.cn

This PDF files includes:
Figures S1-S27.

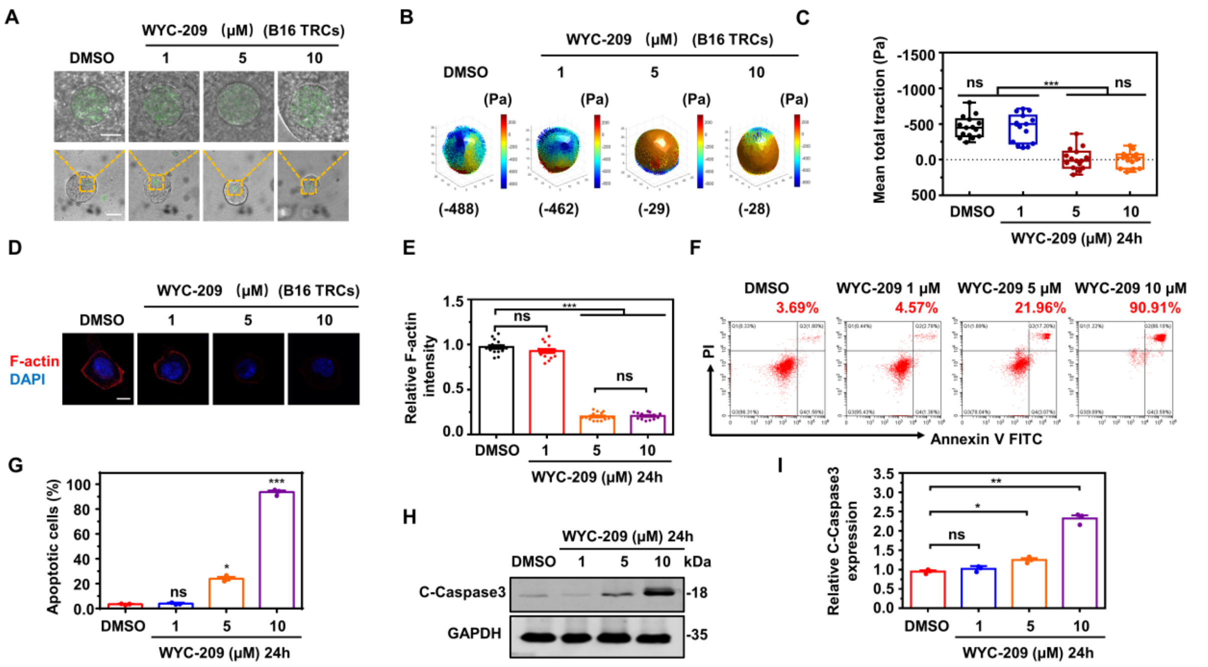

Figure S1. WYC-209 at 5.0 μM completely abolishes tractions and F-actin. Representative images of the elastic round microgels embedded in B16 TRCs colonies (**A**) and of 3D traction force maps of the microgels (**B**) treated with different doses of WYC-209 for 24 h. DMSO: medium with 0.1% DMSO as control. The top images in (**A**) are enlarged images from the respective bottom images. Scale bars in (**A**): top, 10 μm; bottom, 50 μm. (**C**) Quantification data of mean traction forces. Mean ± s.e.m.; n=15 microgels in each condition; three independent experiments. Representative immunofluorescence images (**D**) of F-actin and quantification data of relative F-actin intensity (**E**) of B16 TRCs treated with different doses of WYC-209 for 24 h. Scale bar: 10 μm. Mean ± s.e.m.; n=15 cell samples; three separate experiments. (**F**) Representative images of flow cytometry of B16 TRCs under WYC-209 for 24 h and quantification data of apoptotic cells ratio (**G**) analyzed by FITC-Annexin V and PI apoptosis detection kit. Apoptotic cells (%) =100% - (Annexin-V$^-$ PI$^-$)%. Mean ± s.e.m.; three independent experiments. (**H**) Representative western blot images of Cleaved-Caspase3 and quantification of relative protein expressions (**I**) of B16 TRCs under WYC-209. Mean ± s.e.m.; three independent experiments; one-way ANOVA testing followed by a Tukey post-hoc test when appropriate was used for statistics. $^{*}P<0.05$; $^{**}P<0.01$; $^{***}P<0.001$; ns=not significantly different.

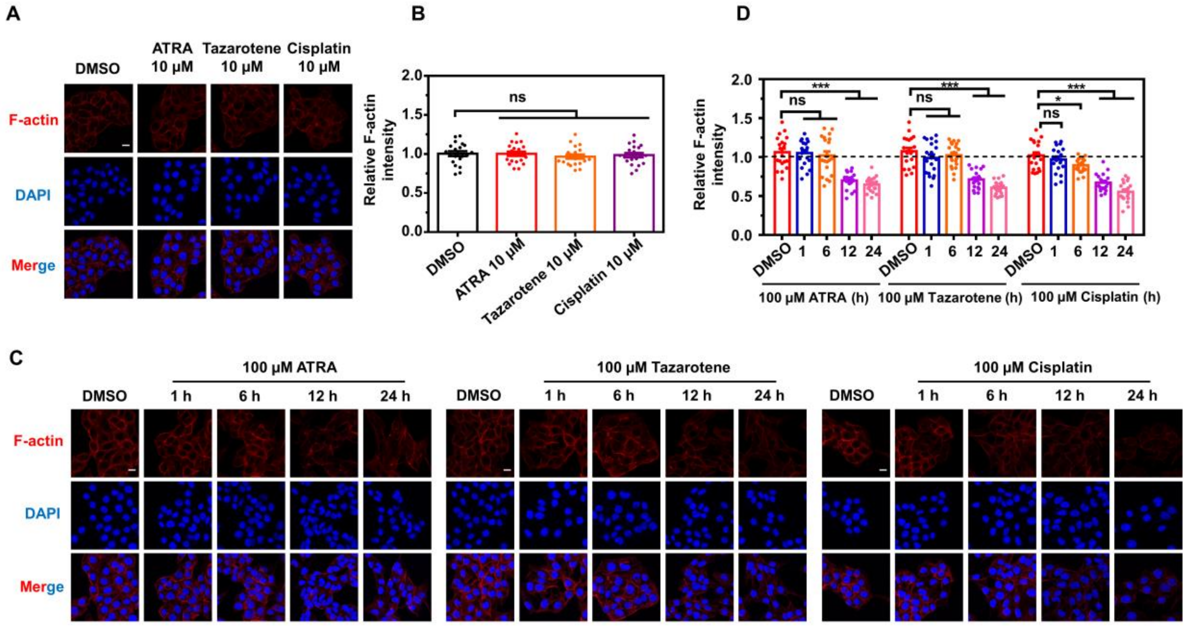

Figure S2. Conventional anticancer drugs at high concentrations (100 μM) induce F-actin depolymerization. Representative immunofluorescence images (**A**) of F-actin and quantification data of relative F-actin intensity (**B**) of B16 TRCs groups treated with 10 μM ATRA, 10 μM Tazarotene or 10 μM Cisplatin for 24 h. Scale bar: 10 μm. Mean ± s.e.m.; n=20 cell samples; three separate experiments. Representative immunofluorescence images (**C**) of F-actin and quantification data of relative F-actin intensity (**D**) of various B16 TRCs groups treated with 100 μM ATRA, 100 μM Tazarotene or 100 μM Cisplatin for different time points. DMSO as control. Scale bar: 10 μm. Mean ± s.e.m.; n=20 cell samples; three separate experiments. One-way ANOVA testing followed by a Tukey post-hoc test when appropriate was used for statistics. $^{*}P<0.05$; $^{***}P<0.001$; ns=not significantly different.

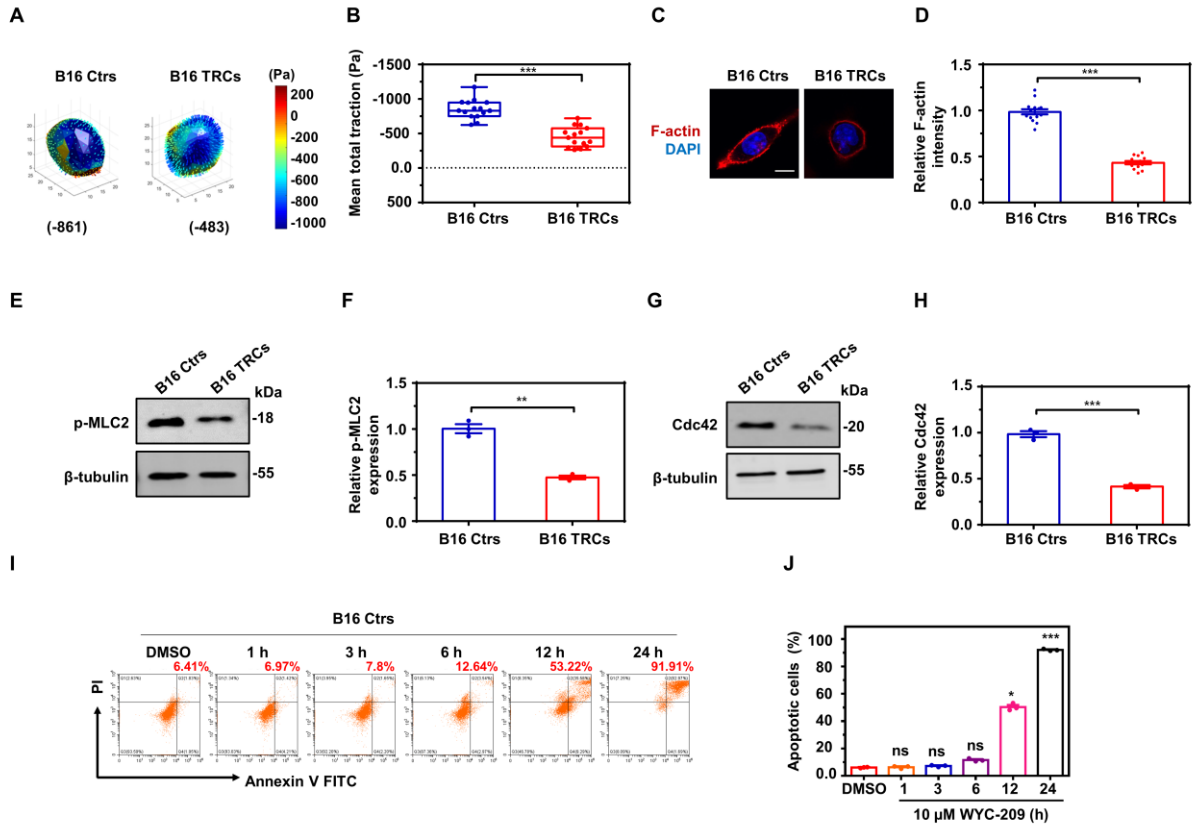

Figure S3. TRCs express lower levels of Cdc42 and tractions than differentiated melanoma cells. Representative images of 3D traction force maps (**A**) of the elastic round microgels embedded in B16 TRCs and B16 Ctrs and quantification data (**B**) of mean tractions. Mean ± s.e.m.; n=15 microgels in each condition; three independent experiments. Representative image (**C**) of F-actin stained by phalloidin (red) and nucleus by DAPI (blue). Scale bar: 10 μm. (**D**) Relative F-actin intensity. Mean ± s.e.m.; n=15 cell samples; three separate experiments. Representative western blot image (**E**) of p-MLC2 and quantification data (**F**) of relative p-MLC2 expression. Mean ± s.e.m.; three separate experiments. Representative western blot image (**G**) of Cdc42 and quantification data (**H**) of relative Cdc42 expression in B16 Ctrs and B16 TRCs. Mean ± s.e.m.; three separate experiments. Representative images of flow cytometry data (**I**) and quantification data of apoptotic cells (**J**) of B16 Ctrs treated with 10 μM WYC-209 for different time points. Apoptotic cells (%) = 100% - (Annexin-V^{-} PI^{-}) %. Mean ± s.e.m.; three independent experiments. B16 TRCs: B16-F1 cells were cultured in 90-Pa fibrin gels for 3 days and then re-cultured on 2D 90-Pa fibrin gels. B16 Ctrs: B16-F1 cells cultured on 2D rigid dishes. Student's t-test (only two data groups compared) and one-way ANOVA testing followed by a Tukey post-hoc test when appropriate was used for statistics. $^{*}P$<0.05; $^{**}P$<0.01; $^{***}P$<0.001; ns=not significantly different.

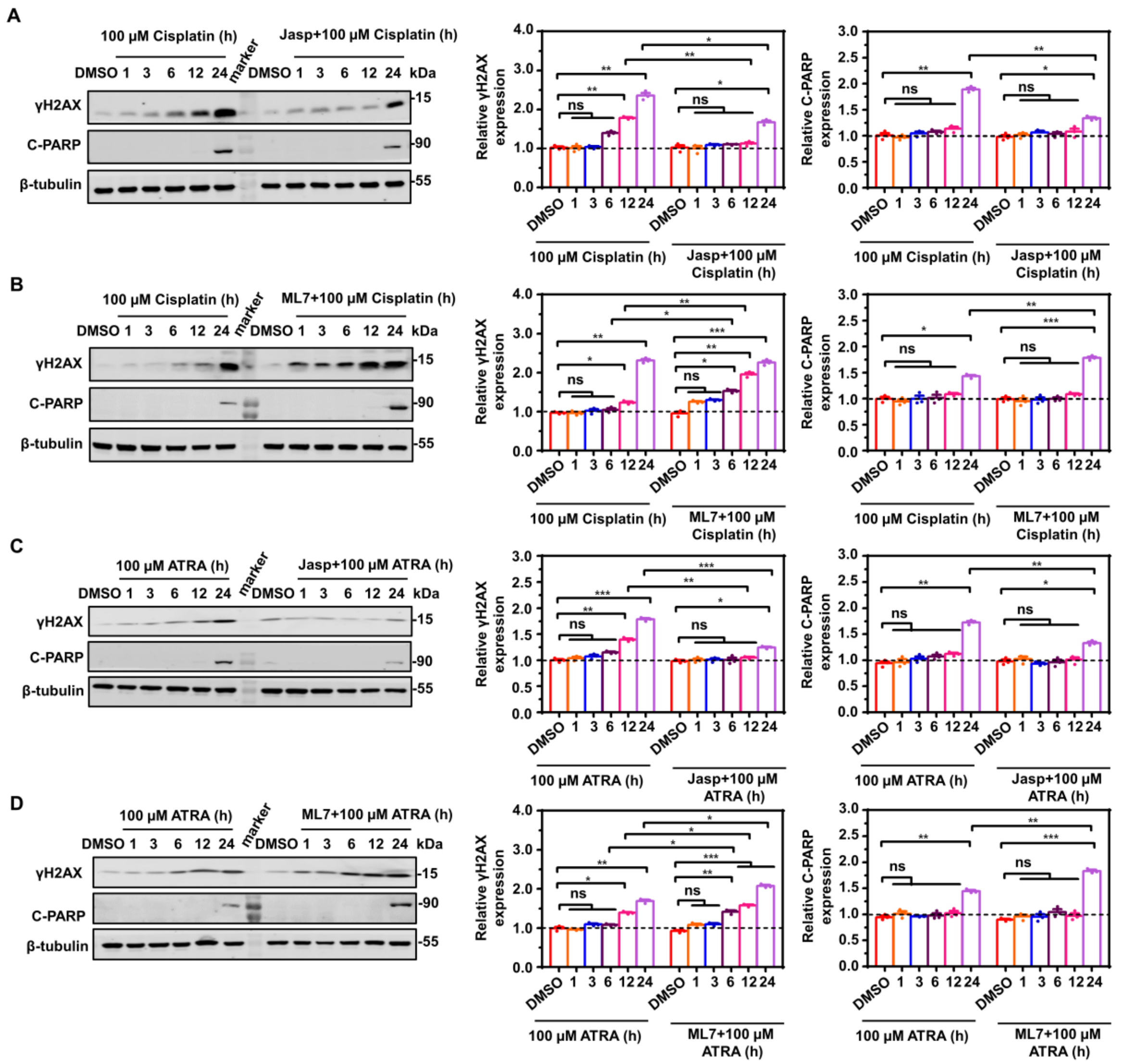

Figure S4. Modulation of F-actin and tractions regulates TRC apoptosis by conventional anticancer drugs. (**A**) Representative western blot images (**left**) of γH2AX and Cleaved-PARP expression and quantification data (**right**) of relative proteins levels of B16 TRCs groups treated with 100 μM Cisplatin with Jasplakinolide or not for different time points. Mean ± s.e.m.; three separate experiments. (**B**) Representative images (**left**) of γH2AX and Cleaved-PARP expression and quantification data (**right**) of relative proteins levels of B16 TRCs groups treated with 100 μM Cisplatin with ML7 or not. Mean ± s.e.m.; three separate experiments. (**C**) Representative western blot images (**left**) of γH2AX and Cleaved-PARP expression and quantification data (**right**) of relative proteins levels of B16 TRCs groups treated with 100 μM ATRA with Jasplakinolide or not. Mean ± s.e.m.; three separate experiments. (**D**) Representative western blot images (**left**) of γH2AX and Cleaved-PARP expression and quantification data (**right**) of relative proteins levels of B16 TRCs groups treated with 100 μM ATRA with ML7 or not. Mean ± s.e.m.; three separate experiments. One-way ANOVA testing followed by a Tukey post-hoc test when appropriate was used for statistics. $^{*}P<0.05$; $^{**}P<0.01$; $^{***}P<0.001$; ns=not significantly different.

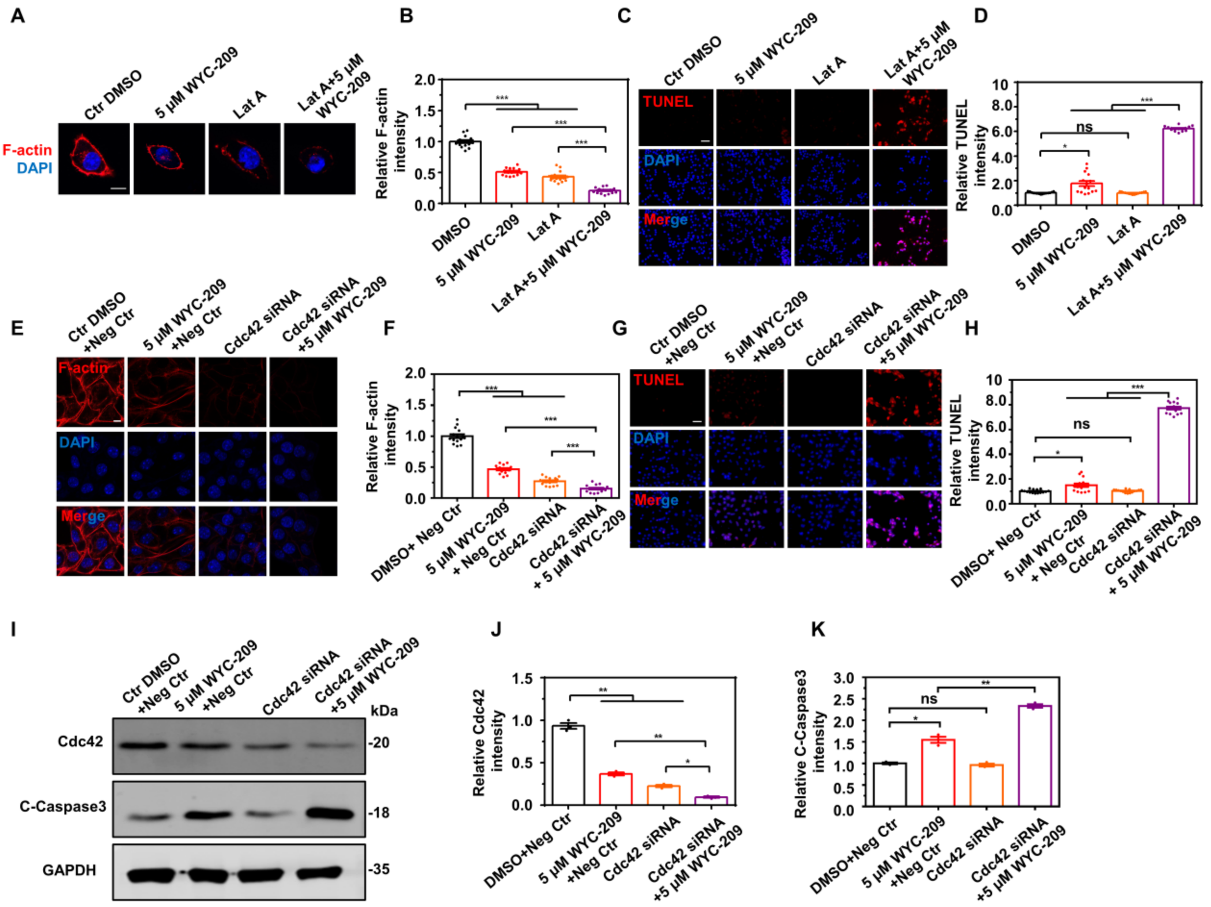

Figure S5. Depleting Cdc42 upregulates retinoid-induced differentiated tumor cell apoptosis via downregulating F-actin. Representative images (**A**) and relative intensity (**B**) of F-actin in B16 Ctrs groups. DMSO: medium with 0.1% DMSO; 5.0 μM WYC-209: 5.0 μM WYC-209 for 24 h; Lat A: 0.3 μM for 0.5 h; Lat A+5.0 μM WYC-209: pretreated with Lat A 0.3 μM for 0.5 h, washed out and treated with 5.0 μM WYC-209 for 23.5 h. Scale bar: 10 μm. Mean ± s.e.m.; n=15 cell samples; three separate experiments. Representative images (**C**) and quantification of relative intensity (**D**) of TUNEL intensity of B16 Ctrs with same treatments as above. Scale bar: 50 μm. Mean ± s.e.m.; n=15 samples; three separate experiments. (**E-K**) B16 Ctrs transfected with scrambled siRNA (Neg Ctr) or Cdc42 siRNA under DMSO (Dimethyl sulfoxide, 0.1%) or 5.0 μM WYC-209 for 24 h. Representative images (**E**) of F-actin and quantification data (**F**) of relative F-actin intensity. Scale bar: 10 μm. Mean ± s.e.m.; n=15 cells; three separate experiments. Representative images (**G**) and quantification (**H**) of relative intensity of TUNEL staining. Scale bar: 50 μm. Mean ± s.e.m.; n=15 cells; three separate experiments. Representative western blot images (**I**) of Cdc42 and Cleaved-Caspase3 and quantification of relative proteins levels (**J, K**). Mean ± s.e.m.; three independent experiments; one-way ANOVA testing followed by a Tukey post-hoc test when appropriate was used for statistics. $^{*}P<0.05$; $^{**}P<0.01$; $^{***}P<0.001$; ns=not significantly different.

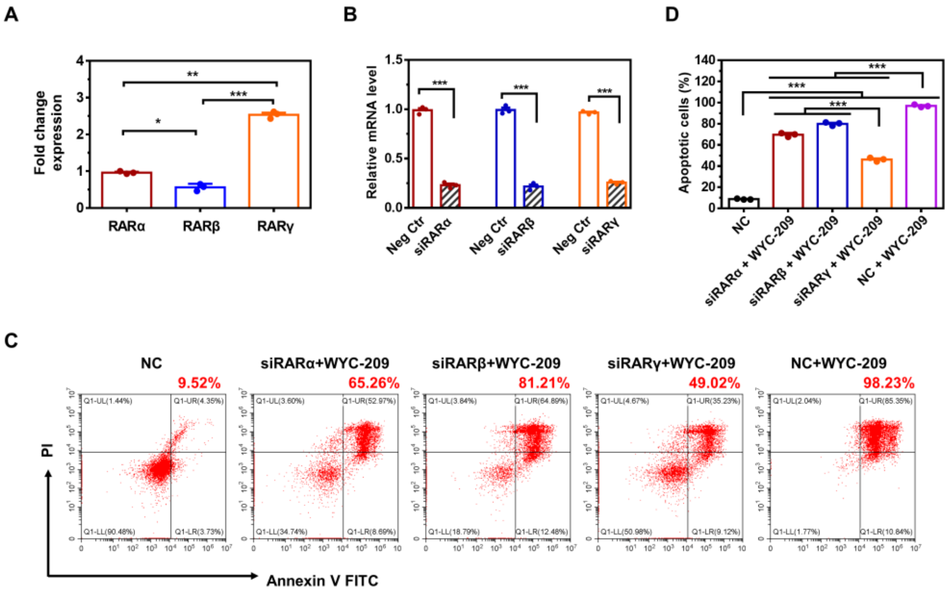

Figure S6. RARγ is the dominant binding target for WYC-209. (**A**) RARs mRNA expressions in B16 TRCs, normalized with GAPDH. Mean ± s.e.m.; three separate experiments. (**B**) Quantification data of RARs mRNA level expression in B16 TRCs after silencing RARs expression. Neg Ctr: B16 TRCs transfected with scrambled siRNA; siRARα: B16 TRCs transfected with siRARα; siRARβ: B16 TRCs transfected with siRARβ; siRARγ: B16 TRCs transfected with siRARγ. Mean ± s.e.m.; three separate experiments. Representative images of flow cytometry data (**C**) and quantification data of apoptotic B16 TRCs ratio (**D**) induced by 10 μM WYC-209 after RARα, RARβ or RARγ silencing treatments. Apoptotic cells (%) = 100% - (Annexin-V$^-$ PI$^-$) %. Mean ± s.e.m.; three separate experiments; Student's t-test (only two data groups compared) and one-way ANOVA testing followed by a Tukey post-hoc test when appropriate were used for statistics. $^{*}P<0.05$; $^{**}P<0.01$; $^{***}P<0.001$.

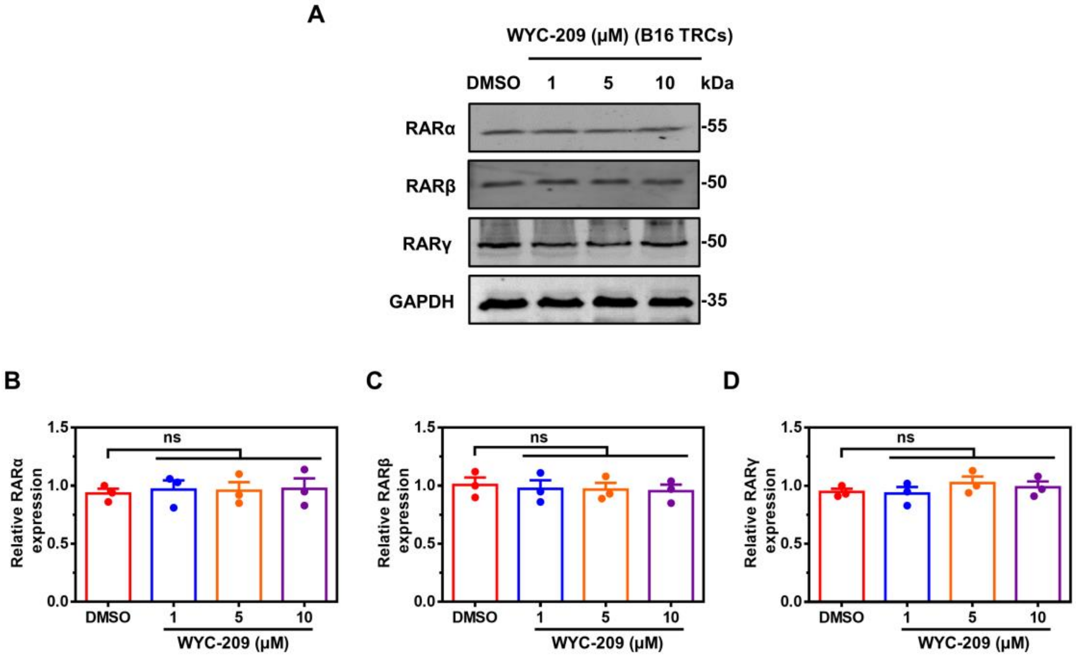

Figure S7. Retinoid WYC-209 does not change RAR expression levels. (**A-D**) Representative western blot images (**A**) and quantification levels (**B-D**) of B16 TRCs of RARα, RARβ and RARγ expression after various concentrations of WYC-209 treated for 24 h. Mean ± s.e.m.; three separate experiments; one-way ANOVA testing followed by a Tukey post-hoc test when appropriate was used for statistics. ns= not significantly different.

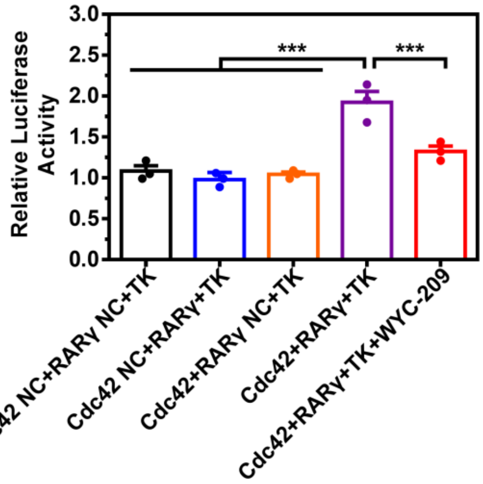

Figure S8. Reporter assays show that RARγ regulates Cdc42 expression via binding to Cdc42 promoter. The plasmids for RARγ overexpression (pcDNA3.1-GFP-RARγ), RARγ NC (pcDNA3.1-GFP-RARγ-NC), and promoter reporter Cdc42 (GPL3-Basic-Cdc42), Cdc42 NC (GPL3-Basic-Cdc42-NC) and TK (pRL-TK) were constructed by GenePharma Corporation (Suzhou, China). Plasmids were transiently transfected into B16 TRCs. After 48 h culture, B16 TRCs were treated with 10 μM WYC-209 for 1 h or not and then the luciferase activities were measured by a Dual Luciferase Reporter Gene Assay Kit according to manufacturer's instructions (RG027, Beyotime, China). Mean ± s.e.m.; three separate experiments. One-way ANOVA testing followed by a Tukey post-hoc test when appropriate was used for statistics. $^{***}P<0.001$.

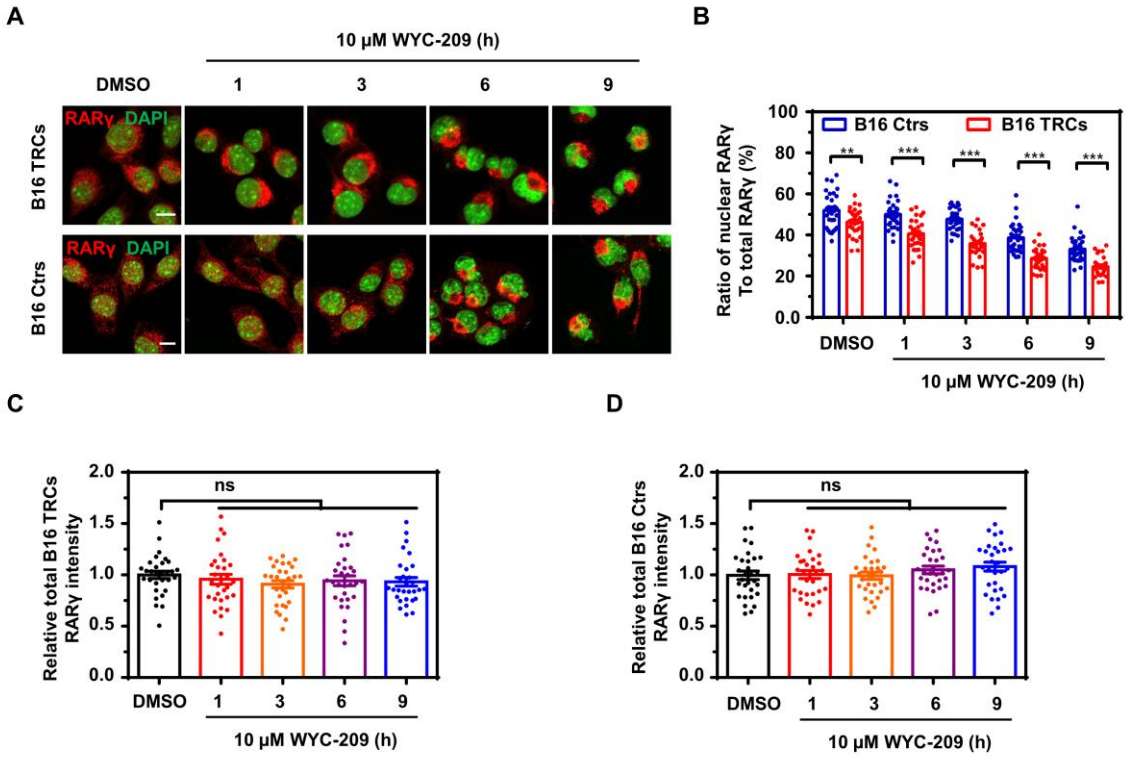

Figure S9. WYC-209 induces transfer of RARγ of TRCs into cytoplasm faster than that of differentiated tumor cells. (**A**) Representative images of nuclear location of RARγ in B16 Ctrs and B16 TRCs treated with 10 μM WYC-209 for various time points. B16 TRCs: B16-F1 cells were cultured in 90-Pa fibrin gels for 3 days and then re-cultured on 2D 90-Pa fibrin gels. B16 Ctrs: differentiated B16-F1 cells cultured on 2D rigid dishes. RARγ was stained by antibody (red) and nucleus by DAPI (green). Scale bar: 10 μm. (**B**) Quantification data of ratio of nuclear RARγ to total RARγ in (**A**). Mean ± s.e.m.; n=30 cells in each condition; three independent experiments. Quantification data of relative total RARγ levels of B16 TRCs (**C**) and control B16 cells (**D**) in (**A**). Mean ± s.e.m.; n=30 cells in each condition; Student's t-test (only two data groups compared) and one-way ANOVA testing followed by a Tukey post-hoc test when appropriate was used for statistics. $^{**}P<0.01$; $^{***}P<0.001$; ns= not significantly different.

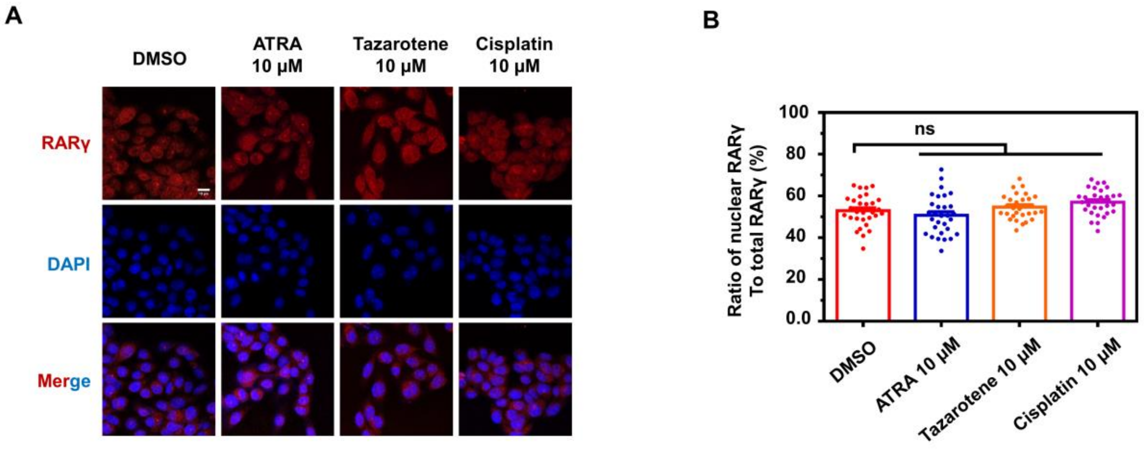

Figure S10. Conventional anti-cancer drugs at low concentration (10 µM) do not induce RARγ translocation. Representative immunofluorescence images (**A**) of RARγ and quantification data of ratio of nuclear RARγ to total RARγ (**B**) of various B16 TRCs groups treated with 10 µM ATRA, 10 µM Tazarotene or 10 µM Cisplatin for 24 h. Scale bar: 10 µm. Mean ± s.e.m.; n=30 cell samples; three separate experiments; one-way ANOVA testing followed by a Tukey post-hoc test when appropriate was used for statistics. ns=not significantly different.

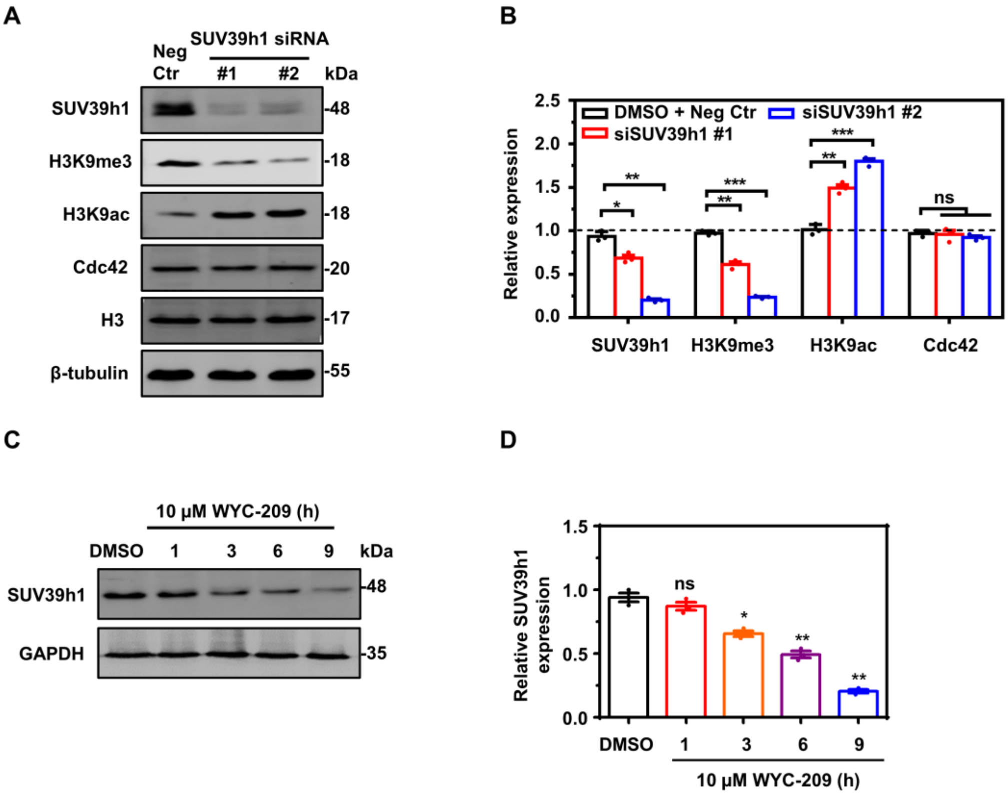

Figure S11. Depleting SUV39h1 decreases H3K9me3 and increases H3K9ac but has no effect on Cdc42. Representative western blot images (**A**) and quantification data (**B**) of SUV39h1, H3K9me3, H3K9ac and Cdc42 expression of TRCs after silencing SUV39h1 expression. Neg Ctr: TRCs transfected with scrambled siRNA; #1 and #2: TRCs transfected with siRNA#1 or #2 of SUV39h1. Mean ± s.e.m.; three separate experiments. Representative western blot images (**C**) and quantification data (**D**) of SUV39h1 after 10 μM WYC-209 treatment for different time points. Mean ± s.e.m.; three separate experiments. One-way ANOVA testing followed by a Tukey post-hoc test when appropriate was used for statistics. $^{*}P<0.05$; $^{**}P<0.01$; $^{***}P<0.001$; ns=not significantly different.

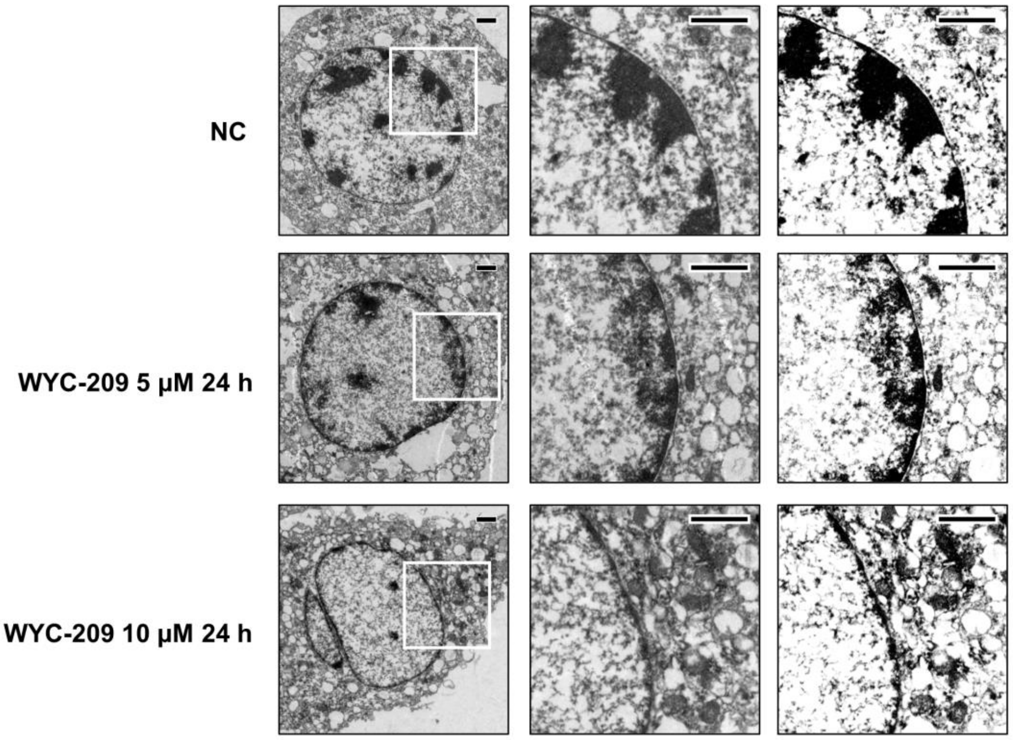

Figure S12. Electron microscopy images show dose-dependent chromatin decondensation by WYC-209. Electron microscopy of B16 TRCs after WYC-209 treatments. White squares on left panel represent area selected for detail on middle panel. Threshold images are shown on the right panel. Note the all scale bars: 1 μm.

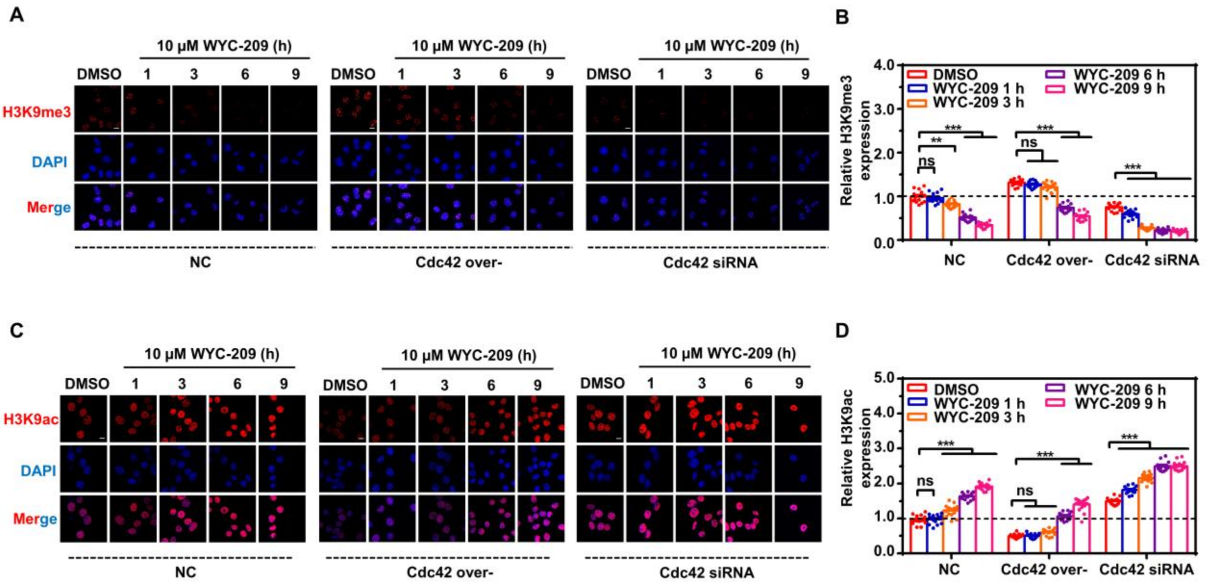

Figure S13. WYC-209 inhibits chromatin condensation by controlling H3K9me3 and H3K9ac via Cdc42. Representative immunofluorescence images (**A**) of H3K9me3 and H3K9ac (**C**) expression and quantification data of relative H3K9me3 (**B**) and H3K9ac (**D**) level of various B16 TRCs groups treated with 10 μM WYC-209 for different time points. Various B16 TRCs groups: NC: negtive control group; Cdc42 over-: B16 TRCs transfected with pcDNA3.1-Cdc42 plasmid group; Cdc42 siRNA: B16 TRCs transfected with Cdc42 siRNA group. Scale bar: 10 μm. Mean ± s.e.m.; n=15 cell samples; three separate experiments; one-way ANOVA testing followed by a Tukey post-hoc test when appropriate was used for statistics. $^{**}P$<0.01; $^{***}P$<0.001; ns=not significantly different.

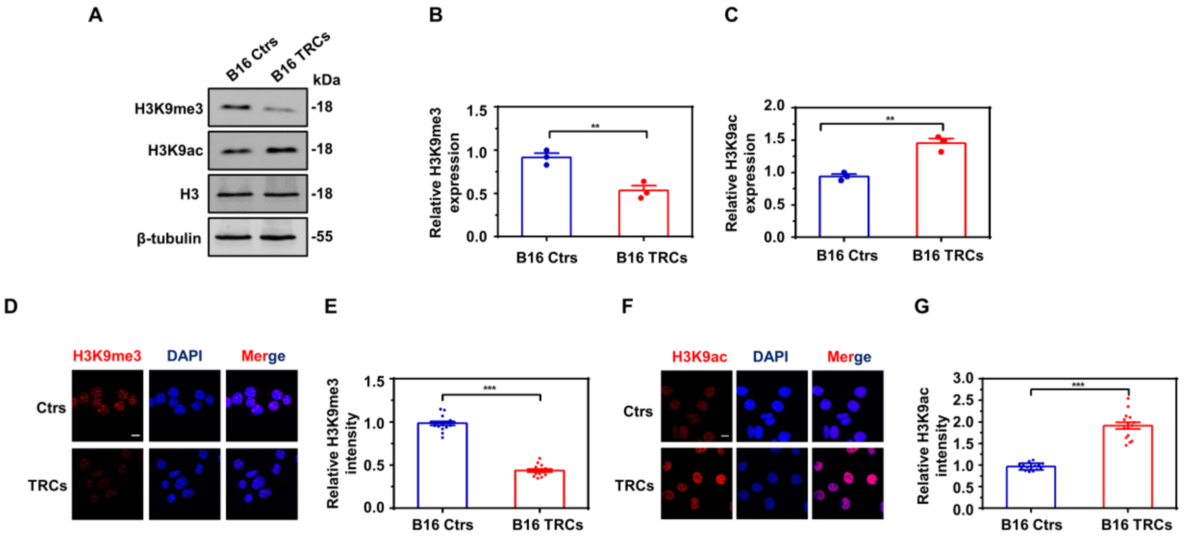

Figure S14. TRCs express lower H3K9me3 and higher H3K9ac than differentiated tumor cells. (**A-C**) Representative western blot images of H3K9me3 and H3K9ac (**A**) and quantification of relative H3K9me3 (**B**) and H3K9ac (**C**) levels of B16 Ctrs and B16 TRCs. Mean ± s.e.m.; three separate experiments. (**D-G**) Representative immunofluorescence images of H3K9me3 (**D**) and H3K9ac (**F**) and quantification of relative H3K9me3 (**E**) and H3K9ac (**G**) intensity of B16 Ctrs and B16 TRCs. TRCs: B16-F1 cells were cultured in 90-Pa fibrin gels for 3 days and then re-cultured on 2D 90-Pa fibrin gels for 4 h. Ctrs: B16-F1 cells cultured on 2D rigid dishes for 4 h. Scale bar: 10 µm. Mean ± s.e.m.; n=15 cell samples; three separate experiments; $^{**}P$<0.01; $^{***}P$<0.001. Two-tails student's t-test was used for statistics.

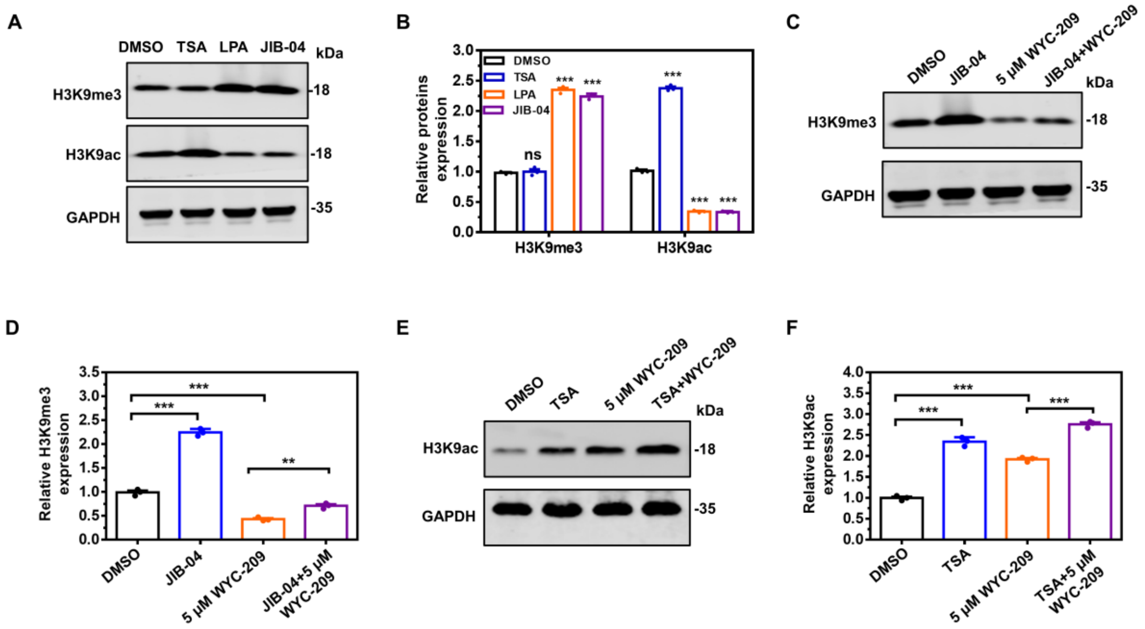

Figure S15. Various drugs regulate H3K9me3 and H3K9ac expression to modulate chromatin condensation levels. Representative western blot images (**A**) and quantification of relative expression (**B**) of H3K9me3 and H3K9ac of B16 TRCs. DMSO: medium with 0.1% DMSO; TSA: 0.5 μM for 4 h; LPA: 10 μM for 4 h; JIB-04: 3.0 μM for 4 h. Mean ± s.e.m.; three separate experiments. Representative western blot images (**C**) and quantification data of relative H3K9me3 expression (**D**) under various treatments. DMSO: medium with 0.1% DMSO; JIB-04: 3.0 μM for 4 h; 5.0 μM WYC-209: 5.0 μM WYC-209 for 24 h; JIB-04+WYC-209: JIB-04, 3.0 μM for 4 h, then washed out them, and treated with 5.0 μM WYC-209 for 20 h. Mean ± s.e.m.; three separate experiments. Representative western blot images (**E**) and quantification of relative H3K9ac (**F**) intensity of B16 TRCs. NC: medium with 0.1% DMSO; TSA: 0.5 μM for 4 h; 5.0 μM WYC-209: 5.0 μM WYC-209 for 24 h; TSA+WYC-209: TSA, 0.5 μM for 4 h, then washed off them, and treated with 5.0 μM WYC-209 for 20 h. Mean ± s.e.m.; three separate experiments. One-way ANOVA testing followed by a Tukey post-hoc test when appropriate was used for statistics. $^{**}P<0.01$; $^{***}P<0.001$. ns= not significantly different.

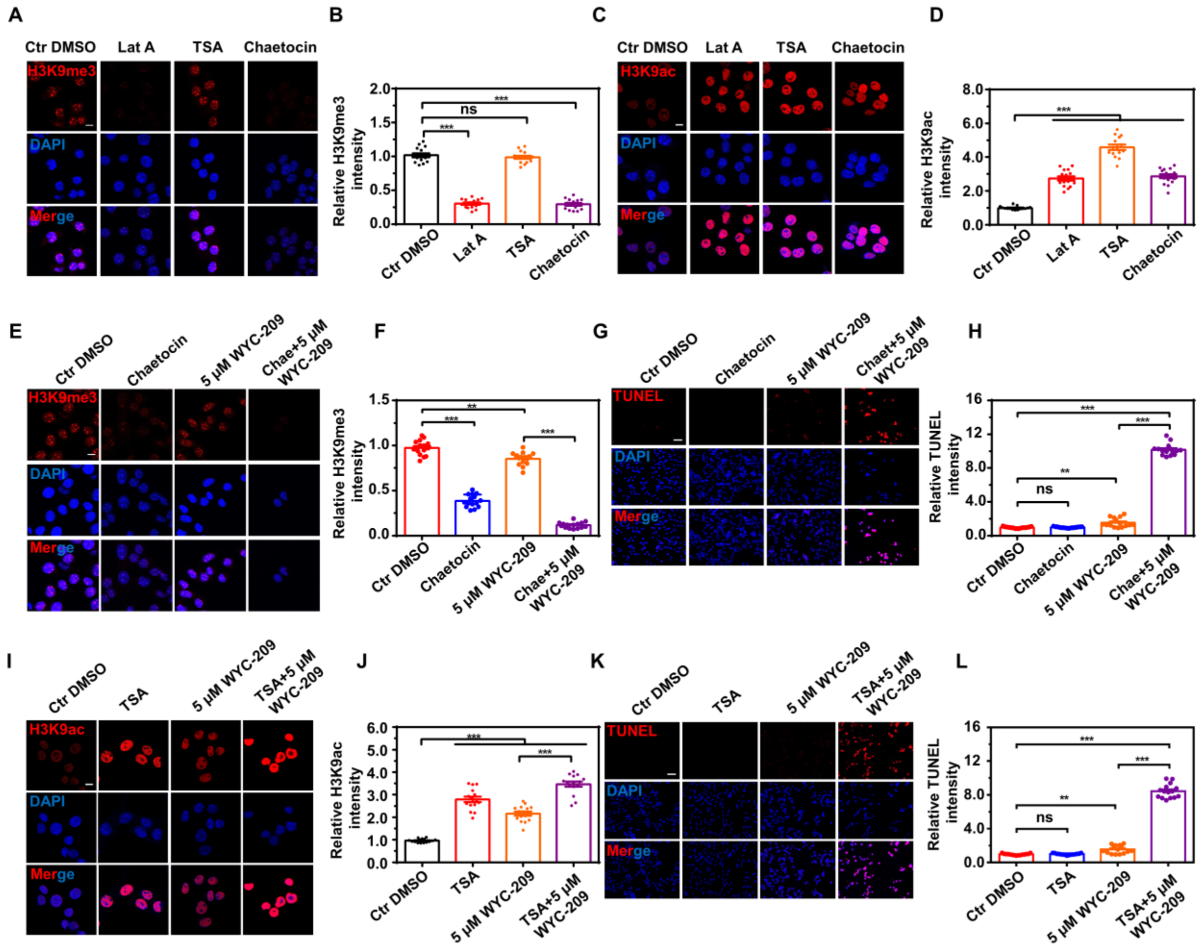

Figure S16. Decondensing chromatin potentiates WYC-209 killing efficiency of differentiated tumor cells. (**A-D**) Representative immunofluorescence images of H3K9me3 (**A**) and H3K9ac (**C**) and quantification of relative intensity (**B, D**) of control B16-F1. DMSO: medium with 0.1% DMSO; Lat A: 0.3 µM for 0.5 h; TSA: 0.5 µM for 4 h; Chaetocin: 0.3 µM for 4 h. Mean ± s.e.m.; n=15 samples; three separate experiments. (**E-H**) Representative immunofluorescence images (**E, G**) and quantification of relative intensity (**F, H**) of H3K9me3 and TUNEL of control B16-F1. DMSO: medium with 0.1% DMSO; Chaetocin: 0.3 µM for 4 h; 5.0 µM WYC-209: 5.0 µM WYC-209 for 24 h; Chae+5.0 µM WYC-209: Chaetocin 0.3 µM for 4 h, then washed off them, and treated with 5.0 µM WYC-209 for 20 h. (**I-L**) Representative immunofluorescence images (**I, K**) and quantification of relative intensity (**J, L**) of H3K9ac and TUNEL of controls with different treatments. DMSO: medium with 0.1% DMSO; TSA: 0.5 µM for 4 h; 5.0 µM WYC-209: 5.0 µM WYC-209 for 24 h; TSA+5.0 µM WYC-209: TSA 0.5 µM for 4 h, then washed off them, and treated with 5.0 µM WYC-209 for 20 h. Mean ± s.e.m.; n=15 samples; three separate experiments. Scale bar in (**A**, **C**, **E**, **I**): 10 µm. Scale bar in (**G**, **K**): 50 µm. One-way ANOVA testing followed by a Tukey post-hoc test when appropriate was used for statistics. $^{**}P<0.01$; $^{***}P<0.001$; ns=not significantly different.

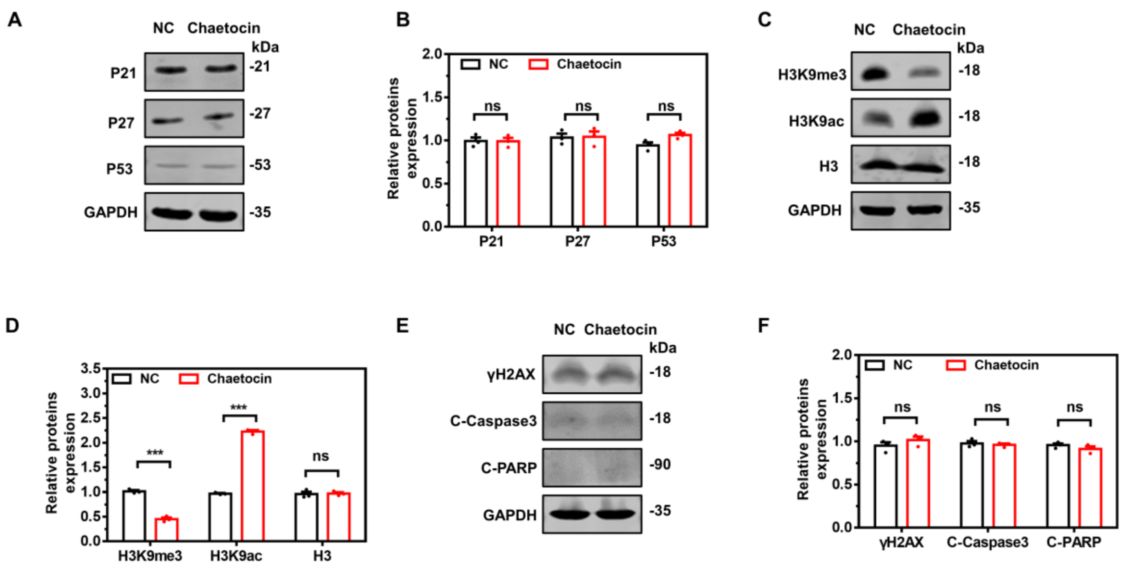

Figure S17. Treating Chaetocin alone induces chromatin decondensation without increasing apoptosis relative proteins expression. Representative western blot images of several proteins (**A, C, E**) of B16 TRCs and quantification of relative proteins expression (**B, D, F**) after various treatments. NC: medium with 0.1% DMSO; Chaetocin: 0.3 μM for 4 h. Mean ± s.e.m.; three separate experiments. $^{***}P<0.001$. ns= not significantly different. Student's t-test was used for statistics.

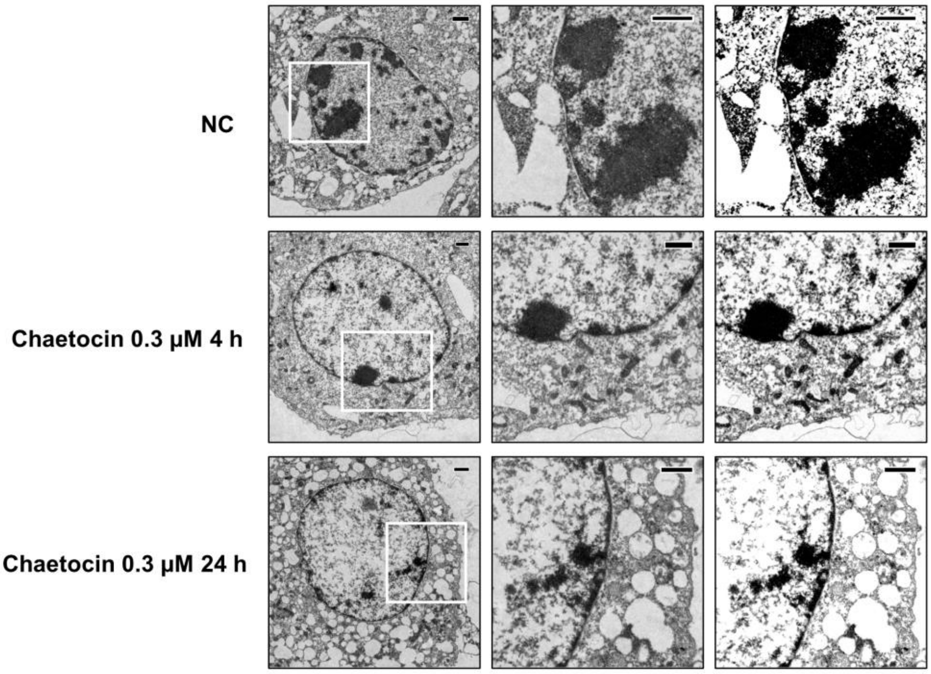

Figure S18. Chaetocin induces chromatin decondensation. Electron microscopy of B16 TRCs after Chaetocin treatments. White squares on left panel represent area selected for enlargement in the middle panel. Threshold images are shown on the right panel. All scale bars: 1 μm.

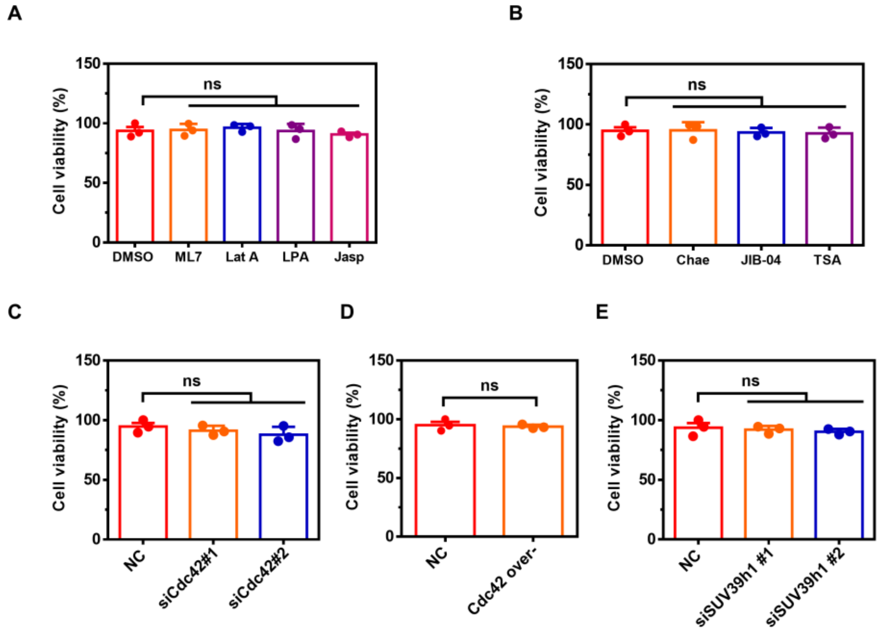

Figure S19. Inhibitors and siRNAs do not change cell viability. (A, B) Different drugs treatments of B16-F1 cell viability measured by MTT. DMSO: 0.1% DMSO. ML-7: 15 μM for 4 h. Lat A: 0.3 μM for 0.5 h. LPA: 10 μM for 4 h. Jasp: 3.0 μM for 4 h. Chae: 0.3 μM for 4 h. JIB-04: 3.0 μM for 4 h. TSA: 0.5 μM for 4 h. Mean ± s.e.m.; three separate experiments. **(C)** B16-F1 cell viability measured by MTT. NC: B16-F1 cells transfected with scrambled siRNA; siCdc42#1: B16-F1 transfected with siRNA#1 of Cdc42; siCdc42#2: B16-F1 transfected with siRNA#2 of Cdc42. (**D**) B16-F1 cell viability measured by MTT. NC: B16-F1 transfected with pcDNA3.1-GFP vector; Cdc42 over-: B16-F1 transfected with pcDNA3.1-Cdc42 plasmid. (**E**) B16-F1 cell viability measured by MTT. NC: B16-F1 cells transfected with scrambled siRNA; siSUV39h1#1: B16-F1 transfected with siRNA#1 of SUV39h1; siSUV39h1#2: B16-F1 transfected with siRNA#2 of SUV39h1. Mean ± s.e.m.; three separate experiments; Student's t-test (only two data groups compared) and one-way ANOVA testing followed by a Tukey post-hoc test when appropriate was used for statistics. ns= not significantly different.

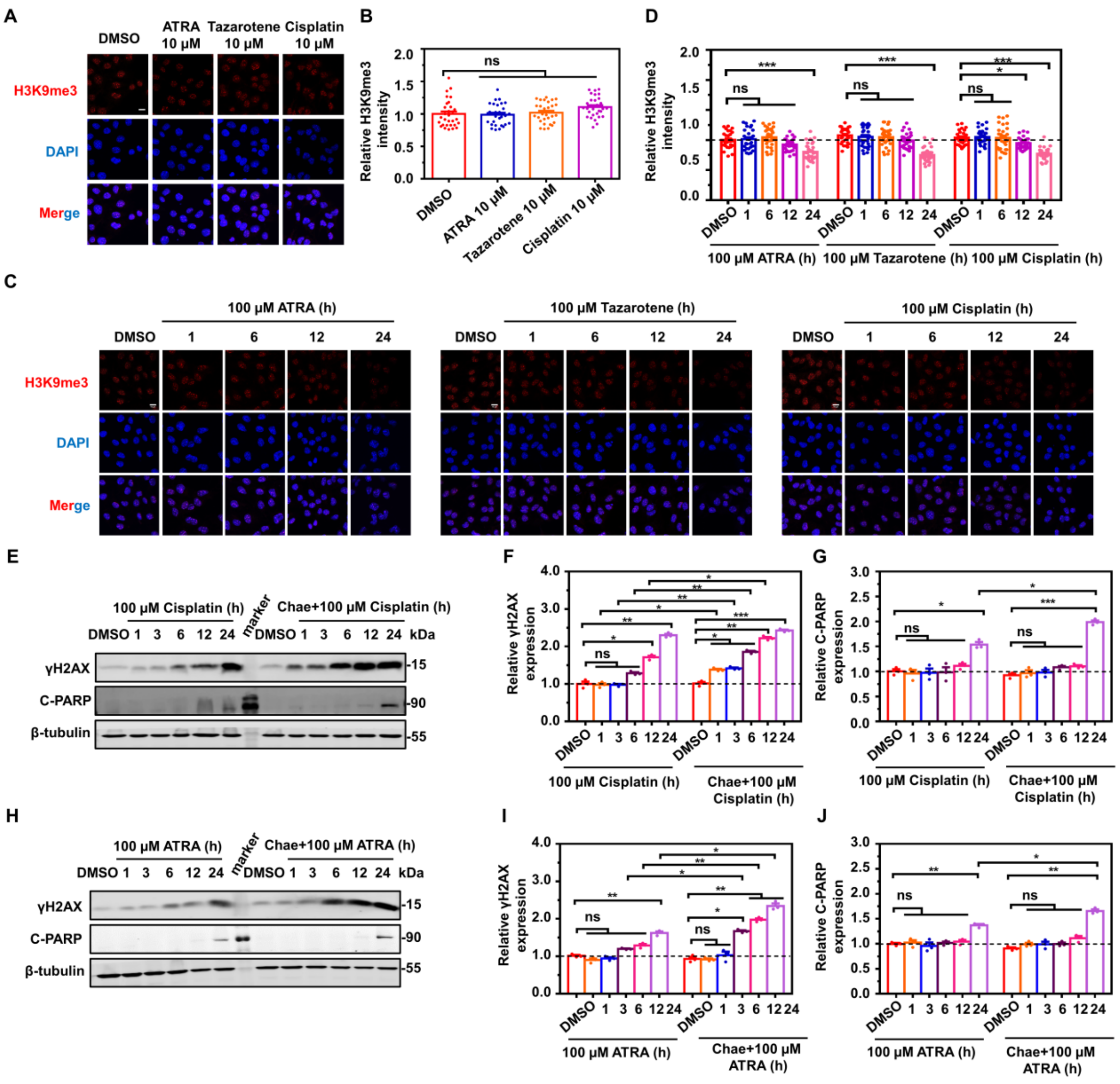

Figure S20. Decondensing chromatin potentiates conventional anticancer drugs-induced DNA damage and apoptosis of TRCs. Representative immunofluorescence images (**A**) of H3K9me3 and quantification data of relative H3K9me3 intensity (**B**) of B16 TRCs treated with 10 μM ATRA, 10 μM Tazarotene or 10 μM Cisplatin for 24 h. Scale bar: 10 μm. Mean ± s.e.m.; n=30 cell samples; three separate experiments. Representative immunofluorescence images (**C**) of H3K9me3 and quantification data of relative H3K9me3 intensity (**D**) of B16 TRCs treated with 100 μM ATRA, 100 μM Tazarotene or 100 μM Cisplatin for different time points. Scale bar: 10 μm. Mean ± s.e.m.; n=30 cell samples; three separate experiments. Representative western blot images (**E, H**) and quantification (**F, G, I, J**) of relative γH2AX and C-PARP expression of B16 TRCs treated with 100 μM Cisplatin or 100 μM ATRA pretreated with or without Chaetocin (0.3 μM for 4 h) for various durations. Mean ± s.e.m.; three independent experiments. One-way ANOVA testing followed by a Tukey post-hoc test when appropriate was used for statistics. $^{*}P$<0.05; $^{**}P$<0.01; $^{***}P$<0.001; ns=not significantly different.

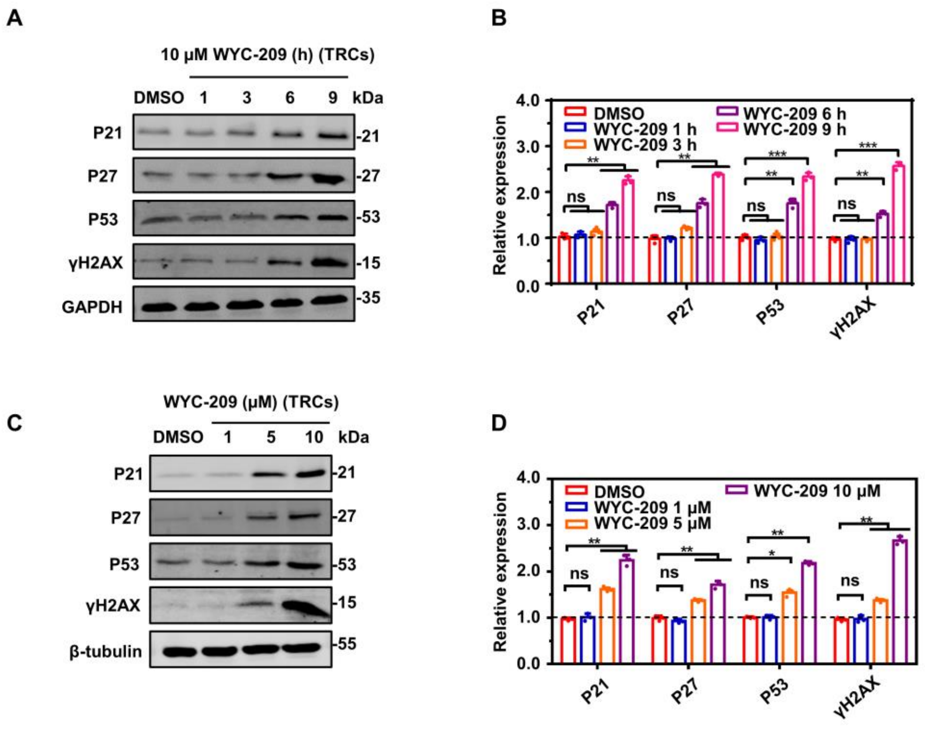

Figure S21. WYC-209 upregulates cell cycle inhibitors and induces DNA damage protein expression. Representative western blot images (**A**) and quantification data (**B**) of P21, P27, P53 and γH2AX under 10 μM WYC-209 treatment for 1, 3, 6, 9 h. Mean ± s.e.m.; three separate experiments. Representative western blot images (**C**) and quantification data (**D**) of P21, P27, P53 and γH2AX under different doses of WYC-209 treatment. Mean ± s.e.m.; three separate experiments; one-way ANOVA testing followed by a Tukey post-hoc test when appropriate was used for statistics. $^{*}P<0.05$; $^{**}P<0.01$; $^{***}P<0.001$; ns=not significantly different.

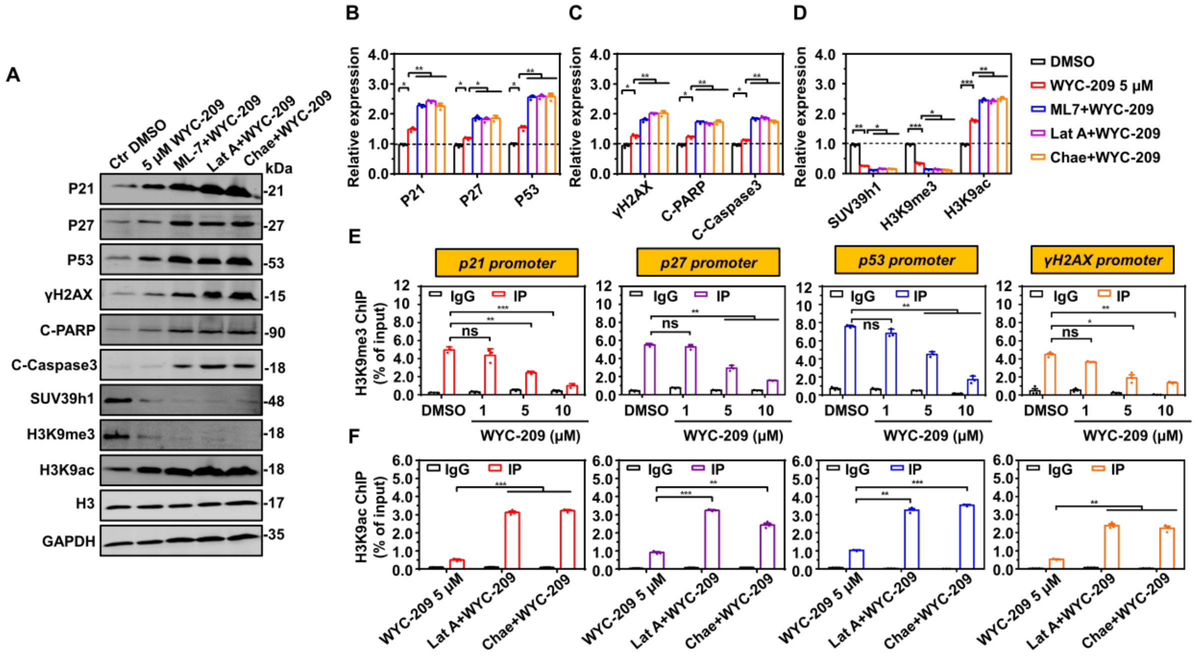

Figure S22. Inhibting F-actin or decondensing chromatin upregulates WYC-209-induced apoptosis and DNA damage. (**A-D**) Representative western blot images (**A**) of P21, P27, P53, γH2AX, Cleaved-PARP, Cleaved-Caspase3, H3K9ac, H3K9me3, SUV39h1 expressions of B16 Ctrs under different treatments. Various treatments: Ctr DMSO: medium with 0.1% DMSO; 5.0 μM WYC-209: 5.0 μM of WYC-209 for 24 h; ML-7+WYC-209: pretreated with ML-7 (15 μM for 4 h) then washed out and treated with 5.0 μM WYC-209 for 20 h; Lat A+WYC-209: pretreated with Lat A (0.3 μM for 0.5 h) then washed out and treated with 5.0 μM WYC-209 for 23.5 h; Chae+WYC-209: pretreated with Chaetocin (0.3 μM for 4 h) then washed out and treated with 5.0 μM WYC-209 for 20 h. (**B-D**) Quantification data of relative proteins expression in (**A**). Mean ± s.e.m.; three independent experiments. (**E**) ChIP assays were performed using normal rabbit IgG (negative control, 2 μg/IP), H3K9me3 antibody (2 μg/IP) on sheared chromatin from 3 million control B16-F1 cells treated with 0.1% DMSO, WYC-209 of 1.0 μM, 5.0 μM, 10 μM for 24 hours. (**F**) ChIP assays were performed using normal rabbit IgG (negative control, 2 μg/IP), H3K9ac antibody (2 μg/IP) on control B16-F1 treated with 5.0 μM WYC-209, 5.0 μM WYC-209+ 0.3 μM Lat A, 5.0 μM WYC-209+ 0.3 μM Chaetocin. (**E, F**) IP DNA relative to input DNA on *P21*, *P27*, *P53*, *γH2AX* promoters were determined by qPCR. Mean ± s.e.m; three independent experiments. One-way ANOVA testing followed by a Tukey post-hoc test when appropriate was used for statistics. $^{*}P< 0.05$; $^{**}P< 0.01$; $^{***}P< 0.001$; ns=not significantly different.

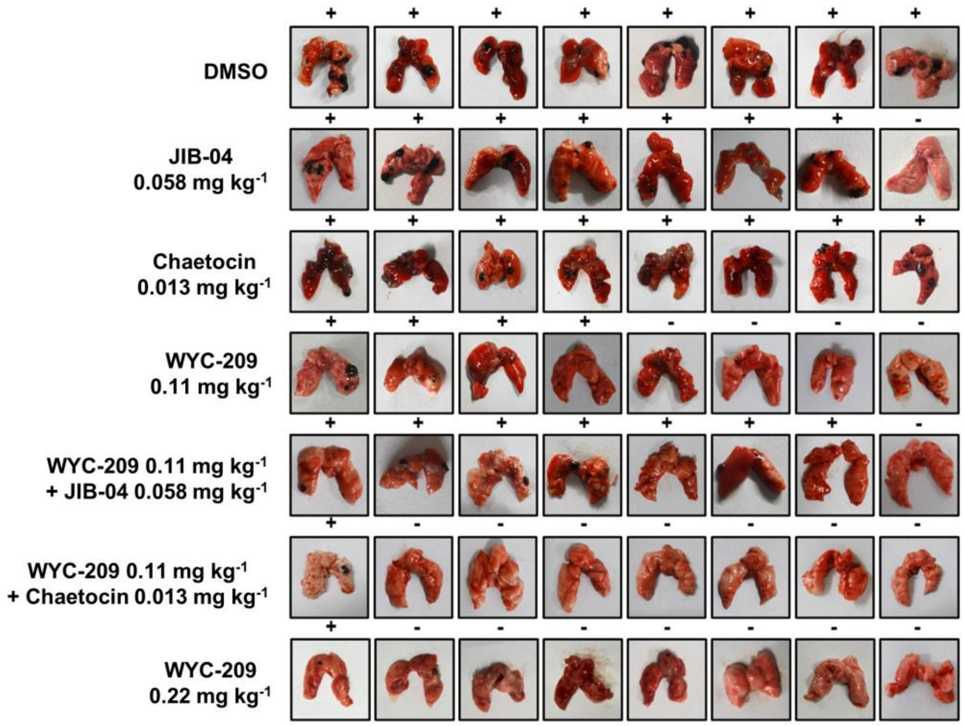

Figure S23. Images of melanoma metastases to the lung from each mouse under various treatments. Images of lungs in Fig.7C at day 30. “+”: lungs with tumor metastases; “-”: lungs without tumor metastases. Images of hematoxylin and eosin (H&E) staining of lung tissues (not shown) from each group are consistent with the anatomical images shown in this figure.

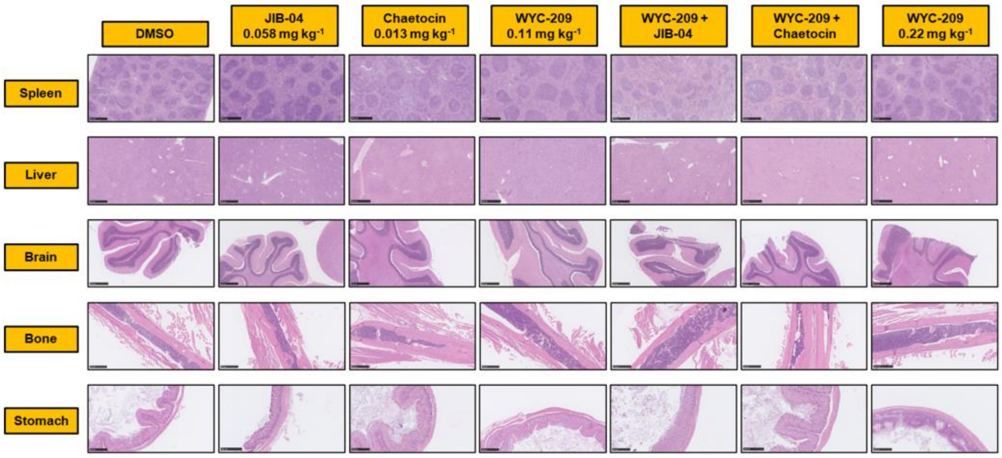

Figure S24. Other mouse organs do not show signs of tumor formation. Representative images of hematoxylin and eosin (H&E)-stained sections of spleen, liver, brain, bone and stomach from C57BL/6 mice intravenously injected with 100,000 TRCs per mouse. The mice were dissected on day 30 or on the day of death to examine signs of metastases. DMSO**,** JIB-04 0.058 mg kg^{-1}, Chaetocin 0.013 mg kg^{-1}, WYC-209 0.11 mg kg^{-1}, WYC-209 0.11 mg kg^{-1} + JIB-04 0.058 mg kg^{-1}, WYC-209 0.11 mg kg^{-1} + Chaetocin 0.013 mg kg^{-1}, WYC-209 0.22 mg kg^{-1}. Mice were injected with TRCs for 5 days and then were treated with 0.1% DMSO or the compounds every two days via intravenous injection. All images were taken at 5X magnification; all scale bars, 500 µm. Three random sections per organ were performed. No tumors are found in any of these organs.

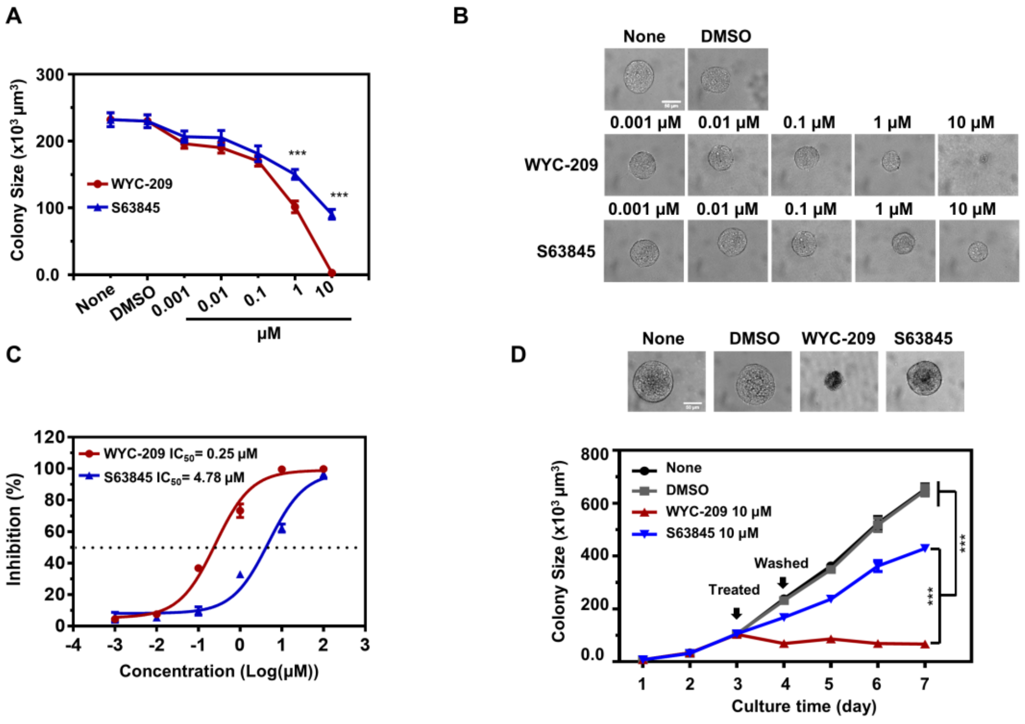

Figure S25. WYC-209 is more potent than S63845 in inhibiting TRC growth. (**A**) Dose-effect curves of WYC-209 and S63845. Colony sizes of B16-F1 on day 5 were quantified. B16-F1 cells were seeded into 90-Pa fibrin gels and treated on day 0. **None**: untreated. **DMSO**: medium with 0.1% DMSO. Mean ± s.e.m.; n=15 samples; three separate experiments. (**B**) Representative images of B16-F1 colonies on day 5. Scale bar, 50 µm. (**C**) B16-F1 TRCs were treated with WYC-209 or S63845 on Day0 and the cell growth inhibition was measured on Day5. Inhibition = 100% × [(Colony size DMSO at Day5 − Colony size DMSO at Day 0) − (Colony size WYC-209 or S63845 at day5 − Colony size WYC-209 or S63845 at Day0)]/(Colony size DMSO at Day5 − Colony size DMSO at Day 0). Graphpad Prism was used to generate IC_{50} curves. Mean ± s.e.m.; three separate experiments. (**D**) B16-F1 cells were cultured in 90-Pa fibrin gels for 3 days and then treated with None (untreated), DMSO (0.1% DMSO), 10 µM WYC-209, or 10 µM S63845 and washed out on day 4. Representative images of B16-F1 colonies on day 7 (top) and quantitative data of colony sizes from day 1 to day 7 (bottom) were shown. Scale bar, 50 µm. Mean ± s.e.m.; n= 15 samples; three separate experiments; Two-tails student's t-test and one-way ANOVA testing followed by a Tukey post-hoc test when appropriate was used for statistics. $^{***}P<0.001$.

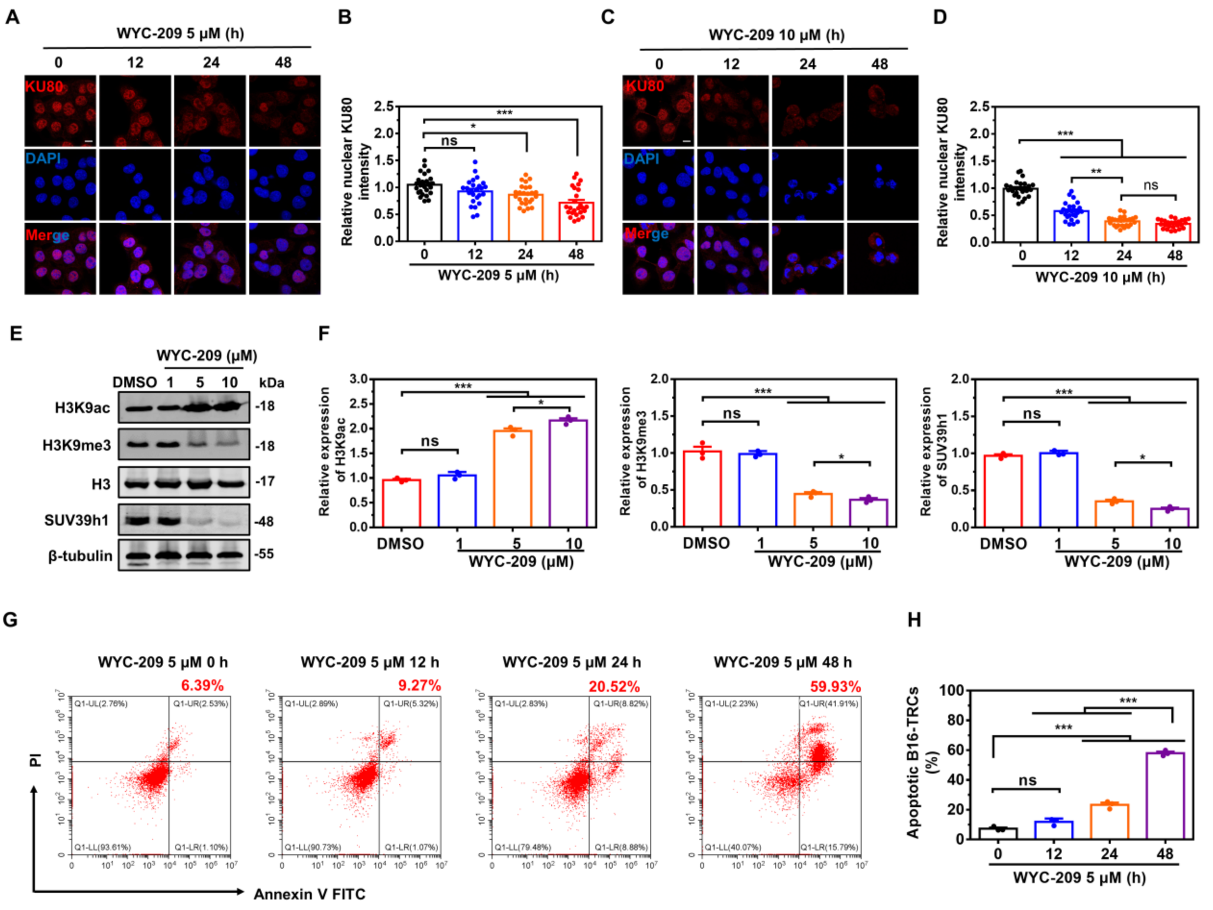

Figure S26. Differences in DNA repair protein and chromatin condensation between 5 μM and 10 μM WYC-209 treatments. (**A**) of Ku80 in B16 TRCs treated with 5.0 μM WYC-209 for various time points. B16 TRCs: B16-F1 cells were cultured in 90-Pa fibrin gels for 3 days and then re-cultured on 2D 90-Pa fibrin gels. Ku80 was stained by antibody (red) and nucleus by DAPI (blue). Scale bar: 10 μm. Quantification data of relative nuclear Ku80 intensity (**B**). Mean ± s.e.m.; n=25 cells in each condition; three independent experiments. Representative images (**C**) of Ku80 in B16 TRCs treated with 10 μM WYC-209 for various time points. Ku80 was stained by antibody (red) and nucleus by DAPI (blue). Scale bar: 10 μm. Quantification data of relative nuclear Ku80 intensity (**D**). Mean ± s.e.m.; n=25 cells in each condition; three independent experiments. **(E, F)** 5.0 μM treatment of TRCs induces less chromatin decondensation than 10 μM WYC-209 treatment. Representative western blot images (**E**) and quantification levels of B16 TRCs of H3K9ac, H3K9me3 and SUV39h1. (**F**) expression after various concentrations of WYC-209 treated for 24 h. (**G, H**) Representative images of flow cytometry data (**G**) and quantification data of apoptotic cells (**H**). B16 TRCs were treated with 5.0 μM WYC-209 for different time points and the cells apoptotic ratio analyzed by FITC-Annexin V and PI apoptosis detection kit. Note that Apoptotic cells (%) = 100% - (Annexin-V^{-} PI^{-}) %. Mean ± s.e.m.; three independent experiments. One-way ANOVA testing followed by a Tukey post-hoc test when appropriate was used for statistics. **P*<0.05; ***P*<0.01; ****P*<0.001; ns= not significantly different.

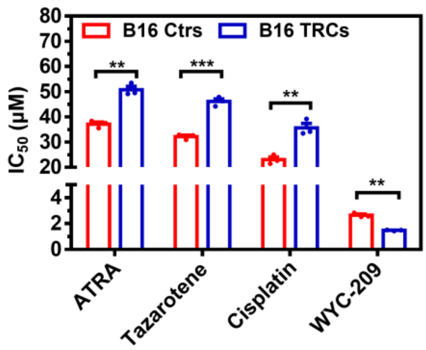

Figure S27. WYC-209 is more potent in TRCs than differentiated tumor cells. IC_{50} of B16 Ctrs (cultured on 2D rigid dishes) and B16 TRCs (cultured in 90-Pa fibrin gels) that were treated with traditional drugs and WYC-209 for 48 h. Mean ± s.e.m.; n=3 samples; three separate experiments; ** P<0.01; *** P<0.001. Two-tails student's t-test was used for statistics (one way?).
